# Divergent Expression Patterns in Two *Vernicia* Species Revealed the Potential Role of the Hub Gene *VmAP2/ERF036* in Resistance to *Fusarium oxysporum* in *Vernicia montana*

**DOI:** 10.3390/genes7120109

**Published:** 2016-12-01

**Authors:** Qiyan Zhang, Ming Gao, Liwen Wu, Yangdong Wang, Yicun Chen

**Affiliations:** 1 State Key Laboratory of Tree Genetics and Breeding, Chinese Academy of Forestry, Beijing 100091, China; zhangqy@caf.ac.cn (Q.Z.); 4862705@163.com (M.G.); wuliwenhappy@163.com (L.W.); 2 Institute of Subtropical Forestry, Chinese Academy of Forestry, Hangzhou 311400, China

**Keywords:** AP2/ERF transcription factor, tung oil tree, *Fusarium* wilt disease, gene expression network, hub gene

## Abstract

Tung oil tree (*Vernicia fordii*) is a promising industrial oil crop; however, this tree is highly susceptible to *Fusarium* wilt disease. Conversely, *Vernicia montana* is resistant to the pathogen. The APETALA2/ethylene-responsive element binding factor (AP2/ERF) transcription factor superfamily has been reported to play a significant role in resistance to *Fusarium oxysporum*. In this study, comprehensive analysis identified 75 and 81 putative *Vf*/*VmAP2*/*ERF* transcription factor-encoding genes in *V*. *fordii* and *V*. *montana*, respectively, which were divided into AP2, ERF, related to ABI3 and VP1 (RAV) and Soloist families. After *F. oxysporum* infection, a majority of AP2/ERF superfamily genes showed strong patterns of repression in both *V*. *fordii* and *V*. *montana*. We then identified 53 pairs of one-to-one orthologs in *V. fordii* and *V. montana*, with most pairs of orthologous genes exhibiting similar expression in response to the pathogen. Further investigation of *Vf*/*VmAP2*/*ERF* gene expression in plant tissues indicated that the pairs of genes with different expression patterns in response to *F. oxysporum* tended to exhibit different tissue profiles in the two species. In addition, *VmAP2*/*ERF036*, showing the strongest interactions with 666 genes, was identified as a core hub gene mediating resistance. Moreover, qRT-PCR results indicated *VmAP2*/*ERF036* showed repressed expression while its orthologous gene *VfAP2*/*ERF036* had the opposite expression pattern during pathogen infection. Overall, comparative analysis of the *Vf*/*VmAP2*/*ERF* superfamily and indication of a potential hub resistance gene in resistant and susceptible *Vernicia* species provides valuable information for understanding the molecular basis and selection of essential functional genes for *V*. *fordii* genetic engineering to control *Fusarium* wilt disease.

## 1. Introduction

Plant growth, development and productivity are strongly affected by fungal pathogen-caused diseases. It is hypothesized that plants have evolved complex signaling pathways and various response mechanisms for overcoming disease via modulation of the expression of defense response genes. Transcription factors are believed to play crucial roles in the transmission of pathogen-derived defense signals to either activate or suppress downstream defense gene expression and also in the regulation of cross-talk among different signaling pathways [1]. The APETALA2/ethylene-responsive element binding factor (AP2/ERF) superfamily is one such transcription factor family that has been reported to a play significant role in resistance to fungal pathogens [2,3].

The AP2/ERF superfamily, one of the largest transcription factor families in plants, is characterized by the AP2 domain, a conserved 60–70 amino acid DNA-binding domain (DBD) that was initially identified in homeotic genes regulating flower development in *Arabidopsis thaliana* [4]. The AP2/ERF superfamily can be divided into ERF, AP2, related to ABI3 and VP1 (RAV) and Soloist families according to the number of AP2 domains and the presence of other conserved motifs [5]. Members with tandem duplicated AP2 domains as well as a single AP2 domain that differs from the ERF AP2 domain are classified as the AP2 family [6], which can be further subdivided into the AP2 and AINTEGUMENTA (ANT) groups [2]. Genes in this family participate in organ-specific growth and developmental regulation [7,8,9,10]. RAV family genes carry a single AP2/ERF domain and an additional B3 DNA-binding domain, which is conserved in other plant-specific transcription factors [5,11]. This family has been shown to mediate gene expression in response to abiotic and biotic stresses [12,13], brassinosteroids [14] and ethylene [15]. Genes with a highly variable gene structure and encoding divergent single AP2 domains are considered members of the Soloist family [5]. ERF family proteins contain a single AP2 domain and are further subdivided into ERF and dehydration-responsive element-binding (DREB) subfamilies [5,16]. Based on their gene structure and phylogeny, members of this gene family have also been further subdivided into ten groups [5,6]. DREB subfamily members typically bind to the dehydration-responsive element/C-repeat element (DRE/CRT, A/GCCGAC) in the promoter region of their target genes [17], and play a crucial role in resistance to abiotic stresses [4]. The ERF subfamily, first discovered in *Nicotiana tabacum* [17], modulates the expression of abiotic stress-responsive genes and pathogenesis-related (PR) genes in disease resistance [18] and in ethylene signaling pathways by recognizing the GCC-box in promoter regions [19]. Many ERF subfamily members also bind to DRE/CRT elements [18,20].

Based on the essential role of the AP2/ERF superfamily in regulating stress responses and developmental processes in plants, various studies have been performed to identify and investigate this family in plants, including *Arabidopsis* [5], rice [5], castor bean [21], Chinese cabbage [22], grape [23], poplar [24], physic nut [25], *Medicago truncatula* [26], Moso bamboo [27], banana [6] and chickpea and pigeonpea [28]. ERF proteins involved in defense against pathogen infection have also been extensively documented [1,2,18]. Overall, *ERF1* overexpression enhances resistance to necrotrophic pathogens such as *Fusarium oxysporum*, *Botrytis cinerea*, and *Plectosphaerella cucumerina* in *Arabidopsis* [29,30]. Indeed, *AtERF14* overexpression had dramatic effects on defense gene expression [31]. It was also found that overexpression of *AtERF5* [32], *AtERF6* [33], *AtERF15* [34], and *ORA59* [35] resulted in increased resistance, whereas the *erf5* *erf6* double mutant and AtERF15-RNAi and ORA59-silenced plants exhibited decreased resistance to *B. cinerea*. In contrast, *Arabidopsis* plants overexpressing *ERF4*, a negative regulator of transcription belonging to group VIII, are more susceptible to *F. oxysporum* than wild type plants [1,30].

*Vernicia fordii* is an important woody non-edible oilseed plant in China. Its seed oil is widely used in industry, such as in inks, lubricants, biodiesel, paints and coatings [36]. However, the wilt disease caused by *F. oxysporum* has greatly adverse effects on its growth and yield. In comparison, *Vernicia montana* is sturdy and shows notable resistance to wilt disease, despite its relatively lower oil production and quality. Given that the ERF family is reported to play significant roles in resistance to *F. oxysporum* [1,29,31] and that *V. montana* is resistant while *V. fordii* is susceptible, it is possible that the AP2/ERF gene family might have undergone divergence with regard to disease tolerance during the evolution of *V*. *fordii* and *V*. *montana* to acquire specific functions. Accordingly, a study on the functional divergence between these resistant and susceptible species may help to clarify the molecular mechanism involved and why one species is more resistant than the other.

To comparatively analyze AP2/ERF superfamily genes in *V*. *fordii* and *V*. *montana,* and identify putative key functional genes in response to *F. oxysporum*, we analyzed the identification, classification, phylogeny and conserved motif distribution among the *Vf*/*VmAP2*/*ERF* superfamily in both *Vernicia* species. Moreover, comparative analysis of expression patterns after *F. oxysporum* infection and tissue-specific expression patterns between the two species as well as interaction networks were performed. The data obtained from this study will help select appropriate genes for further functional characterization and understand the molecular mechanisms of pathogen response of the *Vf*/*VmAP2*/*ERF* superfamily.

## 2. Materials and Methods

### 2.1. Identification of AP2/ERF Superfamily in *Vernicia* Species

AP2/ERF protein sequences of *A. thaliana* were downloaded from the plant TFDB database [37]. An extensive search was performed to identify members of the AP2/ERF transcription factor superfamily based on transcriptome data for *V*. *fordii* and *V*. *montana*. AP2/ERF superfamily protein sequences were analyzed by annotation and the conserved AP2/ERF domains of AP2/ERF protein sequences from *A. thaliana* were used as query sequences to search the transcriptome database. The search was based on BLASTP with a cutoff value of 1 × 10^−5^. Redundant sequences with the same scaffold were removed. The amino acid sequences, corresponding gene sequences, and annotations were retrieved for further analyses. All obtained protein sequences were analyzed to confirm the presence of the characterized AP2/ERF domain using Simple Modular Architecture Research Tool (SMART) [38,39]. The results were reconfirmed using Conserved Domain Database of the National Center for Biotechnology Information (NCBI) [40]. Protein sequences without or with partial AP2/ERF domain were rejected.

### 2.2. Multiple Sequence Alignment of AP2/ERF Superfamily

Multiple sequence alignment of amino acid sequences of the AP2/ERF domain of all putative AP2/ERF superfamily members was generated using BioEdit 7.2.0.0 [41] with its default settings. DNAMAN software (Lynnon BioSoft, San Ramon, CA, USA) was used as a secondary method for sequence alignment and results rechecking. In addition, to compare the evolutionary relationships of AP2/ERF superfamily members in *Vernicia* species and *A. thaliana*, multiple sequence alignment was applied on obtained proteins and *A. thaliana* AP2/ERF superfamily members predicted by Nakano et al. [5].

### 2.3. Phylogenetic Analysis of the AP2/ERF Superfamily

An unrooted phylogenetic tree was constructed with the aligned AP2/ERF superfamily protein sequences of *V*. *fordii* and *V*. *montana* using MEGA5.0 [42] and the maximum likelihood (ML) method, with Poisson correction, pairwise deletion and 1000 bootstrap replicates as parameters. A combined phylogenetic tree between *Vernicia* species and *Arabidopsis* was then constructed, also with MEGA5.0, the ML method and a bootstrap of 1000 replicates.

### 2.4. Motif Recognition of the ERF Family

The conserved motifs outside the AP2/ERF domain in ERF family proteins of *V*. *fordii* and *V*. *montana* were identified using a motif based sequence analysis tool, MEME Suite version 4.10.0 [43], with the following parameters: any number of repetitions of a motif, maximum number of motifs set at 25 and optimum width 6–50 amino acids.

### 2.5. Evolution and Selection of the AP2/ERF Superfamily in *Vernicia* Species

One-to-one orthologous genes of identified *AP2/ERF* genes between *V. fordii* and *V. montana* were predicted using reciprocal-best BLAST-hits (RBH) methods with expected (E)-value threshold of 1 × 10^−5^. The selective pressure on duplicated genes was estimated by calculating the ratio of non-synonymous substitutions (Ka) to synonymous substitutions (Ks) per site using Calculator [44].

### 2.6. Plant Materials and Pathogen Inoculation

*F. oxysporum* was isolated from diseased *V. fordii* in Tianlin County, Guangxi Zhuang Autonomous Region, China, and the strain was routinely cultivated on potato dextrose agar (PDA). Seeding plantlets of the *F. oxysporum*-susceptible cultivar *V. fordii* and the highly resistant cultivar *V. montana* were used. Two-month-old plantlets containing two or three leaves and a healthy root system were selected for these experiments. The sterile roots of chosen plantlets were drilled with a needle and then dipped in the *F. oxysporum* spore suspension of 1 × 10^6^ spores per mL for 30 min. All plantlets infected with the pathogen were replanted in a growth room at 26 °C with a 16 h light/8 h dark photoperiod and 95% relative humidity. The entire root system was harvested and immediately frozen in liquid nitrogen at time points 0, 2, 8 and 13 days post-infection (DPI) for the extraction of total RNA. The drilled roots harvested from the uninfected plants at day 0 as described above were maintained as control. According to the symptoms of wilt disease caused by *F. oxysporum*, the samples from *V. fordii* (F) and *V. montana* (M) were marked as uninfected stage (F0, M0), early stage of infection (F1, M1), middle stage of infection (F2, M2) and late stage of infection (F3, M3). Three independent biological replicates were carried out for the *F. oxysporum* qRT-PCR time course analyses.

### 2.7. RNA Isolation and Expression Analysis

Total RNA was isolated from each sample respectively in *V. fordii* and *V. montana* using the RN38-EASY Spin Plus Plant RNA kit (Aidlab Biotech, Beijing, China) following the manufacturer’s protocol. The quality and concentration of the purified RNA samples were initially evaluated with a 1% agarose gel and a NanoDrop 5000 spectrophotometer (Thermo Fisher Scientific, Waltham, MA, USA) and then the integrity of RNA samples was further confirmed using Agilent 2100 Bioanalyzer (Agilent Technologies, Santa Clara, CA, USA). Samples that had a minimum RNA integrity number (RIN) value of 7.0 were taken for RNA sequencing and qRT-PCR assay. Total RNA (3 µg) was used to synthesize the first strand of cDNA with superscript III reverse transcriptase (Invitrogen, Grand Island, NY, USA) according to the manufacturer’s recommendations.

### 2.8. Quantitative Real-Time PCR Assay

Specific primers of the *AP2/ERF* genes were designed based on the non-conserved regions of corresponding sequence using Primer Premier 5.0 (Premier Biosoft, Palo Alto, CA, USA) with a melting temperature of 58–60 °C, a product size of 150 to 200 bp, and a GC content of 40% to 60%. The primer sequences are listed in Table S4. Three biological replicates from each tissue were pooled into one sample and every sample was analyzed in technical triplicates. The qRT-PCR analysis was performed using the SYBR^®^ Premix Ex Taq TM Kit (TaKaRa, Tokyo, Japan), and all reactions were carried out with an ABI7300 Real-Time quantitative instrument (Applied Biosystems, Foster City, CA, USA). The reaction mixture for qRT-PCR was performed in 20 μL volumes comprised of 2 μL first-strand cDNA, 10 μL 2× SYBR^®^ Premix Ex Taq, 0.4 μL × 10 μM forward primer, 0.4 μL × 10 μM reverse primer, 0.4 μL 50× ROX Reference Dye and 6.8 μL dH_2_O. The amplification conditions were 95 °C for 30 s, followed by 40 cycles at 95 °C for 5 s and then annealing at 60 °C for 31 s. The specificity of the amplifications of each target gene was verified by melting curve analysis. The constitutive gene *ERF1a* was used as an internal control to normalize the gene expression of the chosen transcripts. The relative expression levels of selected genes compared to control were calculated using the 2^−^^△△Ct^ method.

### 2.9. Transcriptome Analysis and Expression Patterns of the ERF Family during Infection

Purified RNA samples were sent to LC-Bio Co., Ltd. (Hangzhou, China) for ribosomal RNA removing, cDNA libraries construction and Illumina sequencing. Transcriptome sequencing libraries were prepared using TruSeqTM RNA Sample Prep Kit (Illumina, San Diego, CA, USA). After all the 24 libraries were constructed, high-throughput sequencing was performed on the IlluminaHiSeq^TM^ 2000 platform. Raw data generated from Illumina sequencing were preprocessed to remove nonsense sequences by discarding the reads with adaptor contamination, masking low-quality reads and removing the reads containing too many unknown bases to produce clean read data. Then, the resulting clean reads were assembled into contigs using the Trinity platform [45]. The contigs were then connected into transcripts according to paired-end information of the sequences. The longest transcriptions from the potential assembled component alternative splicing transcripts were regarded as unigene sequences of our samples. The gene expression levels were normalized to the values of reads per kilobase per million mapped reads (RPKM) using RSEM software [46]. To identify differential expression level and explore *AP2/ERF* genes related to disease resistance in *V. fordii* and *V. montana*, expression patterns of each ERF and DREB subfamily genes were analyzed at different infection stages using transformed RPKM values. A heat map was generated using Multi Experiment Viewer (MeV4) software package [47], with the log2-transformed counts of fold-change values compared with the control (day 0).

### 2.10. Network Analysis of Vf/VmAP2/ERF Superfamily Genes in Response to *F. oxysporum*

A weighted gene coexpression network analysis (WGCNA) with the weighted cut-off value >0.50 was used to identify hub genes highly connected with other genes. The interaction network between *ERF* hub gene and connective genes was constructed with the Cytoscape [48] software. The functional analysis and classification of the connective genes involved in the network were conducted according to orthologous groups (COG) annotation.

## 3. Results

### 3.1. Detection of AP2/ERF Transcription Factors in Two *Vernicia* Species

To study *Fusarium* wilt disease-induced expression of AP2/ERF transcription factors in *Vernicia* species, we identified and characterized *AP2/ERF* genes in the *V. fordii* and *V. montana* transcriptomes using annotation and BLAST with a conserved AP2 domain as the query. After retrieval of redundant genes and analysis for the presence of a characteristic AP2/ERF domain, 92 and 96 candidate *Vf*/*VmAP2*/*ERF* genes encoding full-length and partial AP2/ERF domains were identified in *V. fordii* and *V. montana*, respectively (Table S1). Among them, 75 and 81 putative transcription factors contained the full-length AP2 domain ranging from 531 to 2520 bp and 341 to 2623 bp in length, respectively (Table S1). In *V. montana*, AP2/ERF superfamily proteins were divided into four families, AP2, ERF, and RAV and Soloist (Table 1), according to structural characteristics and the number of AP2/ERF domains. Fourteen *VmAP2*/*ERF* genes were classified into the AP2 family based on the presence of a tandem repeated double AP2/ERF domain; eight of them possessing an extra amino acid in the first AP2 domain were assigned to the ANT subfamily, and other six were assigned to the AP2 subfamily. Two genes predicted to encode one AP2/ERF domain together with one B3 domain were assigned to the RAV family. One gene encoding one AP2/ERF domain was regarded as a member of the Soloist family due to its rather low homology to other AP2/ERF factors. This Soloist family protein shares high similarity with At4g13040, another Soloist protein in *A. thaliana* [5]. The remaining 64 genes encode a single AP2/ERF domain, and these were assigned to the ERF family.

Of the 75 genes identified in *V. fordii*, one and two genes were classified as members of the Soloist and RAV families, respectively. Twelve genes were classified as belonging to the AP2 family, of which six were assigned to the ANT subfamily and the other six to the AP2 subfamily. In addition, 60 genes containing one typical single domain were assigned to the ERF family. Similar to *V. montana*, ERF genes are the most dominant family in *V. fordii*; indeed, at 60 and 64 genes, the ERF family represents 80% and 79.01% of the entire AP2/ERF superfamily in *V. fordii* and *V. montana*, respectively.

### 3.2. Structure and Group-Specific Residues of the AP2 Domains of Vf/VmERF Genes

To determine sequence similarities and phylogenetic relationships among ERF family proteins between *V. fordii* and *V. montana*, multiple sequence alignments were generated using the amino acid sequences of the AP2/ERF domain (Figure S1). Compared to the GCC box-binding domain of *Arabidopsis* ERF1, 25 consensus residues exhibited more than 75% conservation among the 124 ERF proteins from the two species. The alignments showed that residues E16, R26, W28, L29, G30, A38, and A39 are completely conserved among all 124 ERF proteins from the two species. Additionally, another three residues, G4, R6 and R8, are unique to *V. fordii*. In particular, all members of the ERF family in both species contain the WLG motif within the AP2/ERF domain region. Previous studies have shown that A38 in the α-helix, and conserved arginine and tryptophan residues in the β-sheet are essential for AP2/ERF domain DNA binding [49]. All members of the DREB subfamily have a valine residue at position 14, which is crucial for binding to the DRE *cis*-element [50]. Other multiple sequence alignment analyses were performed for the AP2 family between *V. fordii* and *V. montana* (Figure S2). The alignment indicated that residues are more conserved in the first AP2 domain. Compared to the AP2 subfamily, the ANT subfamily contains 11 additional residues in the first AP2 domain.

### 3.3. Phylogenetic Relationships between AP2/ERF Superfamily Transcription Factors

**Figure 1:**
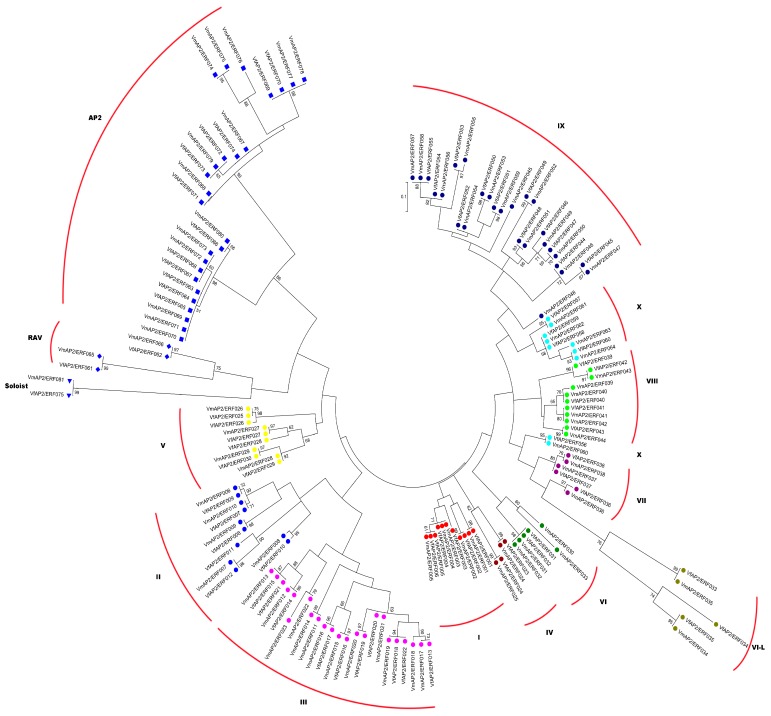
Phylogenetic analysis of APETALA2/ethylene-responsive element binding factor (AP2/ERF) superfamily proteins between *Vernicia fordii* and *Vernicia montana*. An unrooted maximum-likelihood (ML) phylogenetic tree was constructed using MEGA5. Bootstrap value >50% was shown in phylogenetic tree. AP2/ERF proteins were classified into different families and groups.

To determine the evolutionary relationship of AP2/ERF superfamily transcription factors between *V. fordii* and *V. montana*, an unrooted ML phylogenetic tree was constructed based on multiple sequence alignments of the 75 VfAP2/ERF and 81 VmAP2/ERF proteins (Figure 1). According to the phylogenetic tree generated, the 75 and 81 AP2/ERF transcription factors can be divided into 14 distinct clades with well-supported bootstrap values corresponding to the ERF, AP2, RAV and Soloist families. This phylogenetic analysis revealed that members of the RAV family group were classified into separate clades and that ANT subfamily and AP2 subfamily members of the AP2 family group should go separately. Although the Soloist transcription factor contains a single AP2 domain, it clusters together with the AP2 and RAV family. In accordance with the classification of the *Arabidopsis* ERF gene family described by Nakano et al. [5], the 64 ERF family genes with one full AP2 domain from *V. montana* were subdivided into eleven major groups:I through X. No Xb-like gene was found in *V. montana.* Specifically, based on the amino acid similarity of AP2/ERF domains described by Sakuma et al. [16], 39 members containing a conserved alanine at position 14 and an aspartic acid at position 19 were assigned to the ERF subfamily; the 25 remaining genes with a valine and aspartic at the positions 14 and 19, respectively, were identified as possible members of the DREB subfamily. Of these groups, I to IV belong to the DREB subfamily, whereas V to X belong to the ERF subfamily.

Similar to *V. montana*, 60 ERF family genes in *V. fordii* were allocated to groups I through X, though the additional Xb-L group was not found in this species. In addition, 24 genes (groups I–IV) were assigned to the DREB subfamily, and the remaining 36 genes (groups V–X) were assigned to the ERF subfamily. A higher proportion of group IX and a lower proportion of group IV genes were identified in both species, and it is possible that the function of one group can overlap with that of other groups [19]. In comparison to *V. montana*, *V. fordii* has a lower number of ERF genes, and this decrease is observed in almost all the groups.

### 3.4.Distribution of Conserved Motifs outside of the AP2/ERF Domain

**Figure 2:**
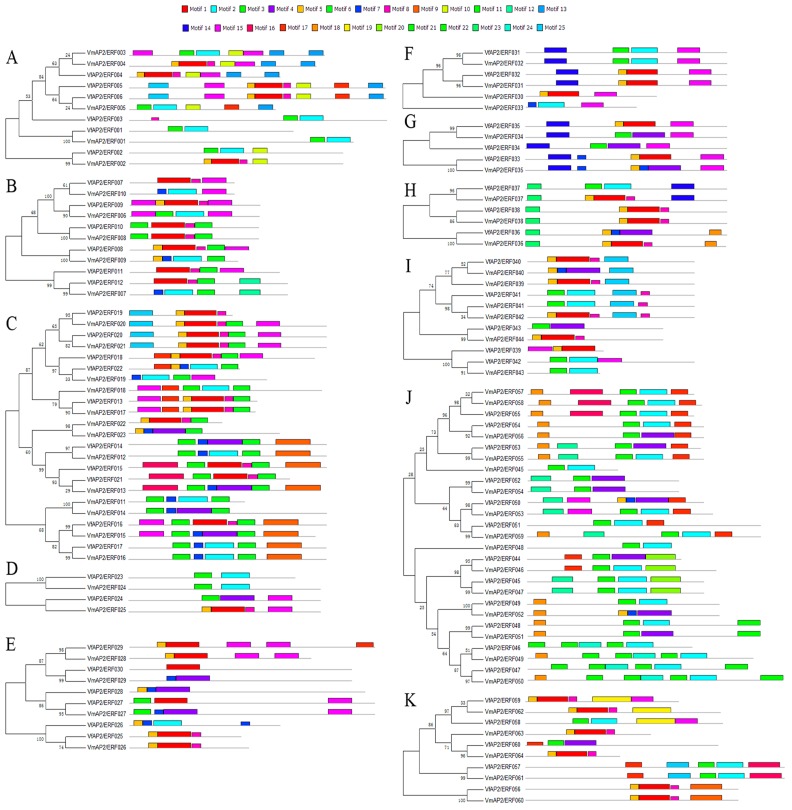
Phylogenetic relationships (**left**) and distribution of conserved motifs (**right**) among the *V. fordii* and *V. montana* ERF proteins from groups I (**A**); II (**B**); III (**C**); IV (**D**); V (**E**); VI (**F**); VI-L (**G**); VII (**H**); VIII (**I**); IX (**J**); and X (**K**). The phylogenetic tree was generated using MEGA5. The motifs (1–25) were identified by MEME, and different motifs are indicated by different colors.

The regions outside the DNA-binding domain in transcription factors generally contain functionally important domains involved in transcriptional activity, protein–protein interactions, and nuclear localization [51]. Such functional sequence motifs are often conserved among members of a subgroup in large families of transcription factors in plants, and proteins with these motifs are likely to have similar functions [52]. To characterize the presence of potential conserved motifs in the ERF family in *V. fordii* and *V. montana*, 124 complete ERF family protein sequences were analyzed using MEME suite version 4.11.1. Overall, 25 conserved motifs were identified and named 1–25 in both species (Table S2). The distribution of these conserved motifs of relevant clades in ERF proteins between *V. fordii* and *V. montana* is illustrated in Figure 2.

The first five motifs and motif 7, as well as motif 11, correspond to the AP2/ERF domain region. The remaining 18 motifs correspond to the conserved region outside of the AP2/ERF domain, and a significant proportion of them are present in specific groups of both species (Figure 2). For the DREB subfamily, motif 10 and motif 13 are specifically distributed in group I. For the ERF subfamily, motif 19 is particularly shared by members of group X, whereas motifs 20, 21, 22 and 24 are specific to group IX. Motif 23 at the N-terminal region is found only in group VII, in each member of this group. Regarding the other motifs, each is shared by members of at least two groups. Overall, motif 15 is present with the highest frequency, in 49 members in all ERF proteins, followed by motif 17 and motif 25. Moreover, ERF proteins clustering in the same clade share one or more similar conserved motifs. Some differences were also identified in conserved motifs between the two species. Motif 15 in groups VIII and X of *V. fordii* was not found in the corresponding groups of *V. montana.* In addition to the common motifs 23 and 14 of group VII in these two species, an extra conserved motif (18) is represented in *V. montana.* This variability might be caused by functional divergences in the ERF genes of these species.

### 3.5. Evolution and Selection of the Vf/VmAP2/ERF Superfamily in *Vernicia* Species

To investigate the evolutionary mechanism of the identified AP2/ERF superfamily in *Vernicia*, phylogeny-based and RBH methods were used to identify possible one-to-one orthologous genes. A total of 53 pairs of putative orthologous genes were found between *V. fordii* and *V. montana* (Table S3).

To verify whether potential selective forces were involved in the divergence of orthologous genes, the ratio of non-synonymous (Ka) and synonymous (Ks) substitution rates was calculated for each pair of orthologous genes (Table S3). In general, Ka/Ks < 1 indicates negative or purifying selection, whereas Ka/Ks > 1 indicates positive or adaptive selection. Ka/Ks = 1 indicates neutral evolution. In total, 15 orthologous genes between *V. fordii* and *V. montana* have exactly the same sequence, with zero Ka and Ks values. With regard to the calculated Ka/Ks ratios of other orthologous genes, 18.42% (7 out of 38 genes) showed Ka/Ks > 1 and 81.58% (31 out of 38 genes) Ka/Ks < 1. Notably, the average value of Ka/Ks was 0.49.

### 3.6. Expression Patterns of Vf/VmERF Genes in *V. fordii* and *V. montana*

**Figure 3:**
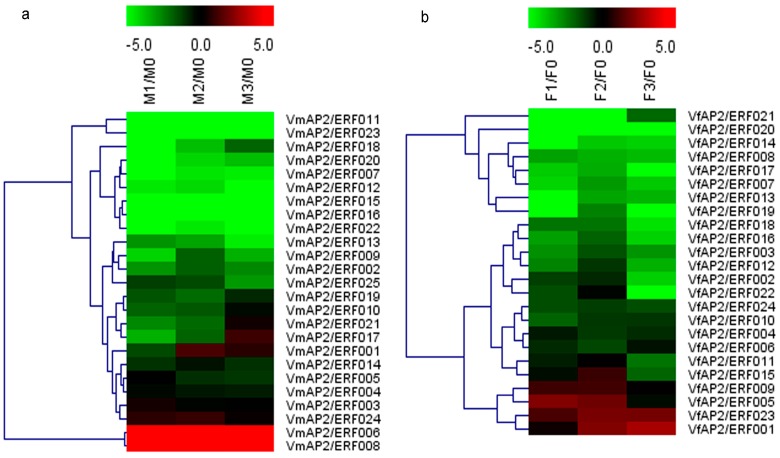
Expression profiles of dehydration-responsive element-binding (DREB) subfamily genes in response to *Fusarium oxysporum* in *Vernicia*, namely (**a**) VmDREB subfamily; and (**b**) VfDREB subfamily. The heat map depicts expression patterns of DREB subfamily genes in *V. fordii* and *V. montana* in response to *F. oxysporum* at four infection stages: 0, uninfected stage; 1, early stage of infection; 2, middle stage of infection; 3, late stage of infection. Color scores were normalized by log2-transformed fold-change values in the treated sample when compared with control sample. The data were generated by averaging the fold change from each of the three biological replicates’ reads per kilobase per million mapped reads (RPKM) values. The color scale is shown at the top. Red represents upregulation and green represents downregulation.

**Figure 4:**
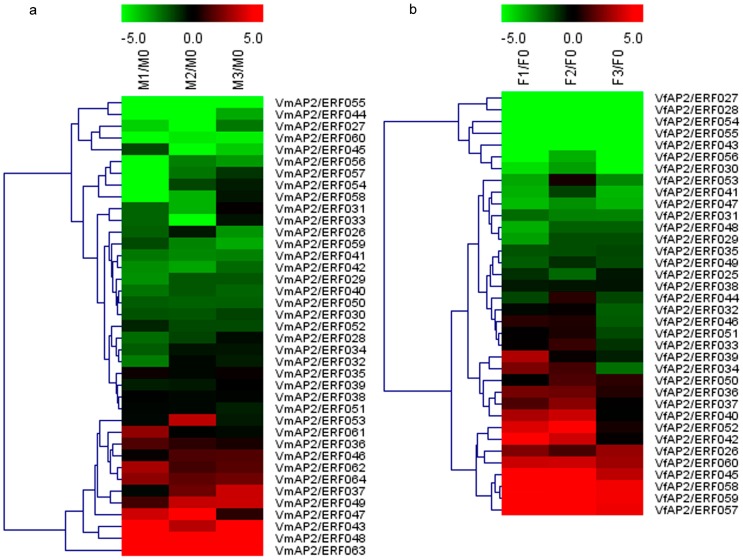
Expression profiles of ERF subfamily genes in response to *F. oxysporum* in *Vernicia*, namely (**a**) VmERF subfamily; and (**b**) VfERF subfamily. The heat map depicts expression patterns of ERF subfamily genes in *V. fordii* and *V. montana* in response to *F. oxysporum* at four infection stages: 0, uninfected stage; 1, early stage of infection; 2, middle stage of infection; 3, late stage of infection. The heat map was generated based on the transformed counts of log2-transformed fold change values in the infected sample compared with the control. The color scale is shown at the top. Red represents upregulation and green represents downregulation.

To compare the expression trend of *ERF* genes between *V. fordii* and *V. montana* and to identify candidate ERFs involved in pathogen resistance, expression patterns of DREB and ERF subfamily genes were examined following pathogen inoculation (Figure 3 and Figure 4). The heat maps were generated based on the normalized RPKM values of the transcriptome data. Compared with the transcript abundance in the control, in general, 59 of 60 *ERF* genes in *V. fordii* responded with more than twofold change in at least one of the three treated stages, and 35 genes showed more than twofold change in expression in all three treatments; of these, 27 (77.14%) ERFs exhibited completely decreased expression but only 7 (20%) increased expression. Similarly, in *V. montana*, 57 of 64 ERF genes responded with more than twofold change in at least one of the three infected stages compared to the uninfected sample, and 36 genes showed more than twofold change in expression in all three treatments. Of these, 26 (72.22%) ERFs exhibited completely decreased expression but only 9 (25%) increased expression. In addition, some ERF family members were present at significant levels in noninduced roots and were repressed to low abundance by pathogen infection in both species. ERFs displaying more than twofold change were present in all phylogenetic groups (Figure S3).

Our analysis also suggested that some *ERF* genes have diverse expression patterns in response to pathogen attack in these two species. With regard to the DREB subfamily (Figure 3), although the number of genes with upregulated or downregulated expression in all the three treatment experiments did not differ greatly in *V. fordii* and *V. montana*, the number of genes displaying transient downregulated expression was different. Compared to the transcript abundance of the control, the expression level of some members in *V. montana* initially dropped and then increased, whereas no gene with this typical expression pattern was found in *V. fordii*. For the ERF subfamily (Figure 4), although the number of *ERF* genes in *V. montana* with sustained decreased expression in the three treatments was higher than in *V. fordii*, the number with sustained increased expression was lower. Regarding transient regulation, family members with initially up- and then downregulated expression were more common in *V. fordii*, with only one member (*VmAP2*/*ERF061*) showing this pattern in *V. montana.*

To compare the expression trends of the 53 pairs of orthologous genes identified between *V. fordii* and *V. montana*, another heat map was generated based on the log2-transformed RPKM values during four infection stages (Figure S4). Our comparative analysis revealed that 34 pairs of orthologous genes exhibited similar expression patterns (*r* > 0.6, *p* < 0.05) in all the four infection stages. Of these, compared to the expression levels of control, 25 pairs of genes exhibited downregulated expression after *F. oxysporum* infection. A small number of orthologous genes showed divergent expression patterns and only five pairs of genes performed completely opposite expression trends (*r* < −0.6, *p* < 0.05) in response to *F. oxysporum*.

### 3.7. Validation of Vf/VmAP2/ERF Gene Expression by qRT-PCR

To validate our transcriptome results, 11 pairs of orthologous *ERF* genes were randomly chosen from the transcriptome data of *V. fordii* and *V. montana* for expression analysis. Specific primers were designed, and qRT-PCR was used to detect *ERF* abundance (Table S4). In general, the expression patterns obtained by the qRT-PCR assay and the transcriptome analysis data were well correlated for these selected genes (Table S5). Although fold changes compared with the control were not similar [6], trends in up- and downregulation of the selected 21 genes (except for *VfAP2*/*ERF045*) were similar, with 12 (*r* > 0.95, *p* < 0.05) having highly similar or even the exact same expression trends in the four infection stages, with nearly identical fold changes.

**Figure 5:**
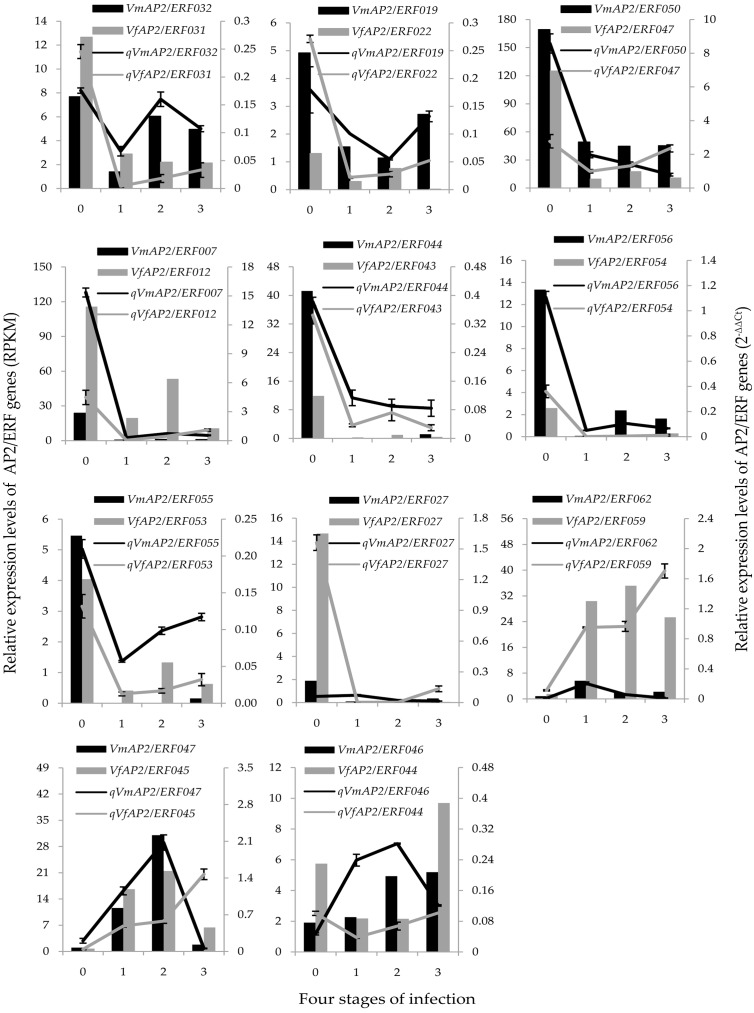
Expression patterns validation of 22 selected *AP2/ERF* genes in root tissue of *Vernicia* during the four infection stages. The *y*-axis represents relative expression level. Primary *y*-axis represents averaged RPKM values from three biological experiments; secondary *y*-axis represents the expression levels calculated from qRT-PCR analysis. Each sample was analyzed by real-time PCR in triplicate. The numbers in the *x*-axis represent the four stages of infection, as follows: 0, uninfected stage; 1, early stage of infection; 2, middle stage of infection; 3, late stage of infection.

The expression profiles of the 22 validated orthologous genes in *V. fordii* and *V. montana* were also evaluated (Figure 5). No obvious regularity was observed for *VmAP2*/*ERF032* expression trend after *F.* *oxysporum* infection. Analysis of the other 10 pairs of *ERF* genes demonstrated that six pairs of *ERF* genes (*VfAP2*/*ERF012*/*VmAP2*/*ERF007*, *VfAP2*/*ERF022*/*VmAP2*/*ERF019*, *VfAP2*/*ERF043*/*VmAP2*/*ERF044*, *VfAP2*/*ERF047*/*VmAP2*/*ERF050*, *VfAP2*/*ERF054*/*VmAP2*/*ERF056*, *VfAP2*/*ERF053*/*VmAP2*/*ERF055*) exhibited similar transcription patterns in response to pathogen attack. Interestingly, compared with the control, the randomly selected orthologous genes with similar expression trends were all downregulated after infection, which further supported our findings, suggesting that suppressed regulation was more common than activated regulation [53]. The other four pairs of ERF genes were differentially and specifically expressed in response to *Fusarium* wilt disease in these two species.

### 3.8. Tissue-Specific Expression Pattern of Vf/VmERF Genes

**Figure 6:**
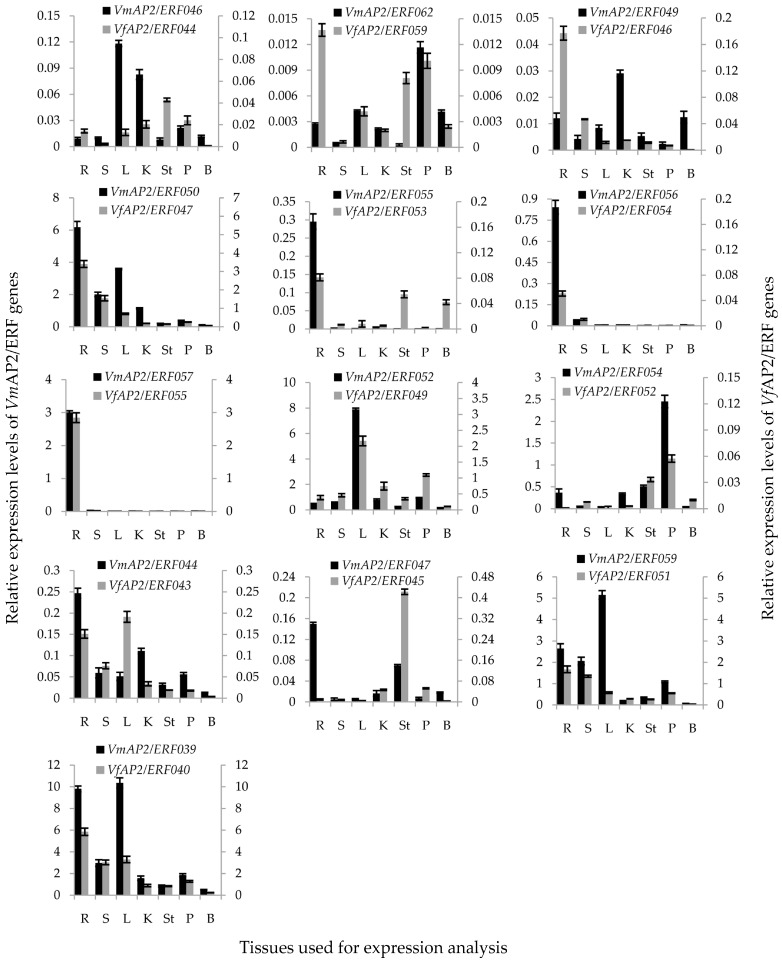
Tissue specific expression analyses of 26 *Vf*/*VmAP2*/*ERF* genes in *Vernicia*. Root (R), stem (S), leaf (L), kernel (K), stamen (St), petal (P) and bud (B) tissues were used for the expression analysis. The *y*-axis represents relative expression level. Primary *y*-axis indicates transcript levels of *VmAP2/ERFs*; secondary *y*-axis indicates transcript levels of *VfAP2/ERFs*. Expression level was analyzed using qRT-PCR in triplicate.

To investigate the tissue-specific expression pattern of *ERF* genes and to further discover genes related to pathogen resistance between *V. fordii* and *V. montana*, qRT-PCR assays were performed on 10 pairs of orthologous genes in group IX and another randomly selected three pairs of genes from the remaining groups. Root, stem, leaf and kernel tissues as well as stamen, petal and bud tissues were used for the expression analysis of these 13 pairs of ERFs (Figure 6).

The expression levels of the selected genes in root tissue revealed that four pairs of *ERF* genes (*VfAP2*/*ERF040*/*VmAP2*/*ERF039*, *VfAP2*/*ERF047*/*VmAP2*/*ERF050*, *VfAP2*/*ERF051*/*VmAP2*/*ERF059* and *VfAP2*/*ERF055*/*VmAP2*/*ERF057*) are expressed to a greater degree between the two *Vernicia* species than the remaining gene pairs (Figure S5). Except for *VfAP2*/*ERF044*/*VmAP2*/*ERF046*, *VfAP2*/*ERF046*/*VmAP2*/*ERF049* and *VfAP2*/*ERF059*/*VmAP2*/*ERF062*, 10 out of 13 pairs of ERFs showed higher transcript levels in root tissue in *V. montana* compared to their corresponding orthologous genes in *V. fordii.* Thus, ERFs in *V. montana* may have higher basal root expression levels than *V. fordii.*

For the 10 pairs of ERFs with higher expression in *V. montana* than *V. fordii*, six showed similar expression patterns in various tissues, and the other four showed different tissue expression patterns between the two species. For the six pairs of genes with similar expression patterns in various tissues, four (*VfAP2*/*ERF047*/*VmAP2*/*ERF050*, *VfAP2*/*ERF053*/*VmAP2*/*ERF055*, *VfAP2*/*ERF054*/*VmAP2*/*ERF056* and *VfAP2*/*ERF055*/*VmAP2*/*ERF057*) were highly expressed in the root compared to other tissues in both species. Moreover, expression of *VfAP2*/*ERF054*/*VmAP2*/*ERF056*, *VfAP2*/*ERF055*/*VmAP2*/*ERF057* and *VmAP2*/*ERF055* was observed only in the root. Together with the similar expression patterns (except for *VfAP2*/*ERF055*/*VmAP2*/*ERF057*), it is possible that these genes share the same function and have a significant role in root development processes or in disease resistance in both species (Figure 5 and Figure 6). *VfAP2*/*ERF055*/*VmAP2*/*ERF057* exhibited similar expression patterns at the first infection stage but divergent patterns at the last two stages. This finding indicates that this pair of orthologous genes may share the same function during initial pathogen infection but that their function begins to differentiate at the last two infection stages via regulation of different signaling pathways and gene interactions (Figure 6, Figure S6).

Other two pairs of ERFs (*VfAP2*/*ERF049*/*VmAP2*/*ERF052*, and *VfAP2*/*ERF052*/*VmAP2*/*ERF054*) showed the highest expression in tissues other than the root in both *V. fordii* and *V. montana*. *VfAP2*/*ERF052*/*VmAP2*/*ERF054* showed specific transcript abundance in petal tissue, and *VfAP2*/*ERF049*/*VmAP2*/*ERF052* were most strongly expressed in leaf tissue. These findings suggest that these genes may have a specific function during floral and leaf development, respectively.

Analysis of the expression level of four pairs of orthologous genes with different tissue expression patterns revealed that *VmAP2*/*ERF044* and *VmAP2*/*ERF047* had the highest transcript abundance in root tissue (0.246 ± 0.011 and 0.150 ± 0.003, respectively) compared to other tissues, though their corresponding orthologous genes *VfAP2*/*ERF043* and *VfAP2*/*ERF045* were specifically expressed in the leaf (0.191 ± 0.012) and stamen (0.423 ± 0.010), respectively. It is possible that *VmAP2*/*ERF044* and *VmAP2*/*ERF047* function in root development and pathogen resistance, but *VfAP2*/*ERF043* and *VfAP2*/*ERF045* are more likely to be involved in the growth and metabolism of other tissues (Figure 5 and Figure 6). Although *VfAP2*/*ERF040* and *VfAP2*/*ERF051* showed the highest expression in root tissue, their corresponding orthologous genes *VmAP2*/*ERF039* and *VmAP2*/*ERF059* were significantly expressed in the leaf in addition to the root. This indicates that these two genes in *V. montana* may control diverse developmental processes in tissues including leaves and roots (Figure 6). Moreover, three out of the four orthologous genes displayed relatively different expression patterns (Figure 6, Figure S6), suggesting their functional divergence in response to wilt disease.

Regarding the three pairs of ERFs with higher expression in *V. fordii* than *V. montana*, all of these genes exhibited tissue-specific expression and different trend patterns in response to *Fusarium* infection (Figure 5 and Figure 6, Figure S6). *VfAP2*/*ERF044* was highly expressed in stamen tissue(0.043 ± 0.001), though the corresponding orthologous gene *VmAP2*/*ERF046* was not significantly expressed in other tissues except leaf (0.118 ± 0.003) and kernel (0.083 ± 0.003). *VfAP2*/*ERF046* and *VfAP2*/*ERF059* were most strongly expressed in root tissue, whereas their corresponding orthologous genes *VmAP2*/*ERF049* and *VmAP2*/*ERF062* showed the highest expression levels in kernel and petal tissues, respectively. Comparing the stable expression level of *VmAP2*/*ERF049* and *VmAP2*/*ERF062* at the four infection stages, *VfAP2*/*ERF046* and *VfAP2*/*ERF059* showed dramatic changes. This result indicates that *VfAP2*/*ERF046* and *VfAP2*/*ERF059* might be essential factors for the response to wilt disease pathogen but that their orthologous genes may primarily participate in kernel and petal development processes, respectively.

### 3.9. Hub Gene VmAP2/ERF036 Mediates Resistance to *F. oxysporum* in *V. montana*

**Figure 7:**
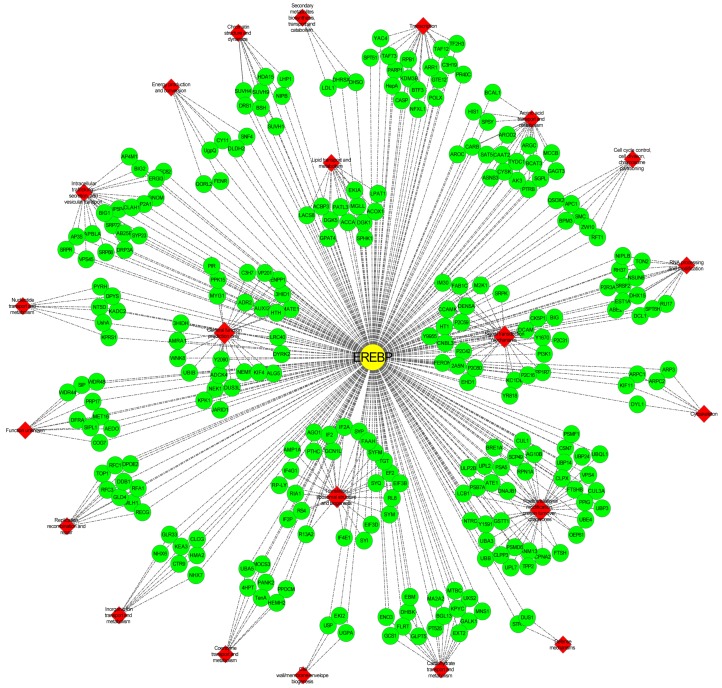
Network analysis of *VmAP2*/*ERF036* gene in response to *F. oxysporum*. The network involves 279 genes exhibiting 550 interactions. The yellow node named ethylene-responsive element binding protein (EREBP) represents the hub gene *VmAP2*/*ERF036*. Green nodes represent the interactive genes with the hub gene. Red diamond nodes represent different clusters corresponding to the putative function of the genes based on clusters of orthologous groups (COG) annotation.

To further understand the interaction between AP2/ERF superfamily genes and other genes in *Vernicia* species, hub genes highly connected with other genes were identified using a WGCNA. Using a weighted cut-off value >0.50, *VmAP2*/*ERF036*, a member of group VII in *V. montana* and having the strongest interactions with 666 genes, was identified as a hub gene. The interaction network between *VmAP2*/*ERF036* and its connected genes was constructed using Cytoscape software (Figure 7). In detail, 316 of the 666 genes were functionally annotated, and their possible functions were predicted and classified (Figure S7). According to COG functional annotations, 278 genes were classified into 22 different clusters involved in RNA processing and modification, amino acid transport and metabolism, carbohydrate transport and metabolism, lipid transport and metabolism, and transcription. The cluster of post-translational modification, protein turnover and chaperones (37; 13.31%) accounted for the largest proportion of the 278 genes, followed by general function (26; 9.35%) and signal transduction mechanisms (26; 9.35%). Of the total, two genes were classified into defense mechanisms, but no genes were assigned to extracellular structures, cell motility and nuclear structure.

**Figure 8:**
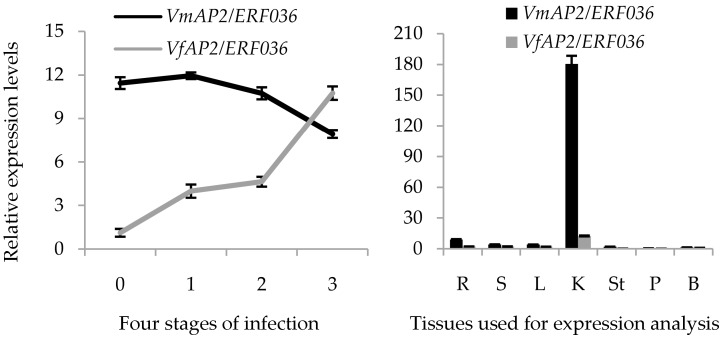
(**left**) Expression trend in response to *F. oxysporum*. The numbers in the *x*-axis represent the four stages of infection, as follows: 0, uninfected stage; 1, early stage of infection; 2, middle stage of infection; 3, late stage of infection. (**right**) Tissue specific expression pattern analysis of *VfAP2*/*ERF036*/*VmAP2*/*ERF036* in root (R), stem (S), leaf (L), kernel (K), stamen (St), petal (P) and bud (B) tissues. The *y*-axis represents relative expression level. Expression level was analyzed using qRT-PCR in triplicate.

To further confirm that *VmAP2*/*ERF036* is a putative key gene involved in pathogen resistance, the expression trend in response to *F. oxysporum* and tissue-specific expression pattern were analyzed (Figure 8). After *F. oxysporum* inoculation, *VmAP2*/*ERF036* showed sustained decreased expression at three stages of infection and exhibited half of the noninduced expression level. Compared to this, its corresponding orthologous genes *VfAP2*/*ERF036* displayed sustained increased expression levels and overtook *VmAP2*/*ERF036* at the third infection stage. Expression patterns in various tissues revealed that both *VfAP2*/*ERF036* and *VmAP2*/*ERF036* showed the highest transcript levels in kernel instead of root tissue. Furthermore, the expression level of *VmAP2*/*ERF036* in kernel tissue is higher than any selected genes in any tissues. However, *VmAP2*/*ERF036* exhibited relative higher expression level in root tissue compared with other selected genes (Figure S6).

## 4. Discussion

In this study, 75 and 81 putative *Vf*/*VmAP2*/*ERF* genes containing a full-length AP2 domain were identified in *V. fordii* and *V. montana*, respectively (Table S1). Compared with other plant species, the number of *AP2/ERF* genes in *Vernicia* species appears to be lower than that in *Arabidopsis* (147) and physic nut (119) (Table 1). The AP2, RAV and Soloist families are of similar sizes and share a highly-conserved sequence with other species, with differences largely induced by changes in the number of ERF family genes. Compared with the 98 *ERF* genes in physic nut, some orthologous genes were not identified in the *Vernicia* transcriptome data. This suggests that these absent ERFs might be expressed in response to stresses other than *Fusarium* wilt disease.

To investigate the evolutionary relationships of AP2/ERF transcription factors between the two *Vernicia* species and *Arabidopsis*, we constructed another unrooted phylogenetic tree of 75 and 81 AP2/ERF family members from *V. fordii* and *V. montana* and 147 AP2/ERF family members from *Arabidopsis* using the conserved AP2 domain (File S1). Most of the AP2/ERF transcription factor members group together with their homologous *Arabidopsis AP2/ERF* gene. In contrast, several ERF groups and subgroups, such as group Xb-L and subgroups IIc, IVb and Xc in *Arabidopsis*, were not found in *V. fordii* and *V. montana*, results that correspond to the study by Hevea [54].

Motif analysis revealed that motif 8, which is specific to group VIII, is identical to the ERF-associated amphiphilic repression (EAR) motif L/FDLNL/F(X)P, which is essential for repression [55]. A previous report has suggested that transcription factors carrying this EAR motif form a complex with co-repressors and histone deacetylases (HDAs), favoring a closed chromatin structure to repress transcription [56]. Motif 15, containing the SP(T/V)SVL sequence, is found in group VI. The SP(T/V)SVL motif was identified as a potential phosphorylation site of mitogen-activated protein (MAP) kinase and/or casein kinase I [5]. Motif 23 is found in the N-terminal region of group VII members, and small blocks of conserved MCCGAI sequences play a role in ethylene transcriptional activation [5,57]. The EDLL motif of ERF family group 9 is necessary for interaction with the activator interaction domain (ACID) of MEDIATOR25 (MED25), which is to date the only co-activator demonstrated to directly interact with ERFs in *Arabidopsis* [30]. It is reported that ERF98 without a functional EDLL motif fails to interact with MED25 and to transactivate reporter genes [58]. In *Vernicia* species, the VmAP2/ERF048 protein was identified as harboring the EDLL motif, indicating that this gene may play a significant role in transactivational activity.

During evolution, gene duplication plays a critical role in the expansion of gene families [59]. Our results about evolution and selection of the 53 pairs of putative one-to-one orthologous genes between *V. fordii* and *V. montana* suggest that a significant proportion of *AP2/ERF* genes have undergone strong purifying selection and that only a small proportion of *AP2/ERF* genes have undergone positive selection. It should be mentioned that *VmAP2*/*ERF036*, with a maximum Ka/Ks ratio of 1.58, shows the most interaction with other genes, as described below (Figure 7).

The global expression analysis of ERFs in *Vernicia* species demonstrated that many *ERF* genes respond to *F. oxysporum* treatment, with decreased transcript abundance being more commonly observed than increased abundance (Figure 3 and Figure 4). This is consistent with the results of Chen et al. [53]. In general, analysis of expression trends revealed that orthologous ERFs largely show rather similar expression patterns in response to wilt disease, though some genes varied between the *Vernicia* species. The differential trends of these ERFs might be due to specific responses to pathogen stress in these species [6]. Combining the differently expressed members of the AP2 family between the two species, it appears that *AP2/ERF* genes mainly exhibit initially up- and finally downregulated expression in *V. fordii*, whereas the opposite trend was more common in *V. montana* after *F. oxysporum* infection (Figure S8).

Disease resistance is a complex biological process involving a series of response genes and signal transduction pathways. Given that genes in group IX have often been linked to defense against pathogen infection (Table 2), tissue-specific expression pattern analysis was performed on 10 pairs of orthologous genes in group IX and another randomly selected three pairs of genes. It suggested that, except for *VfAP2*/*ERF055*/*VmAP2*/*ERF057*, all orthologous genes with similar tissue expression patterns exhibited similar expression trends in response to wilt disease in *V. fordii* and *V. montana*. Orthologous genes with different expression trends in response to wilt disease were more likely to exhibit different tissue expression patterns between the two species (Figure 5 and Figure 6, Figure S6). This difference in expression patterns may have resulted from function differentiation with regard to *F. oxysporum* resistance. Given that *V. montana* is resistant and *V. fordii* susceptible and that *F. oxysporum* invades the roots, it can be concluded that genes having more important functions in *V. montana* roots compared to *V. fordii* are more likely to be critical for controlling the pathogen response.

Among all the interacting genes shown in Figure 7, leucine-rich repeat (LRR) receptor-like serine/threonine-protein kinases (RLPs), which play significant roles in inducing the primary immune response via interaction with fungal effectors such as chitin and xylanase [65], were noteworthy. In addition, components of mitogen-activated protein kinase (MAPK) signal cascades, MAPK and WRKY transcription factor genes were identified in this network. Combined with previously described perspectives [30], it can be hypothesized that RLPs recognize *F. oxysporum* infection and then trigger the MAPK defense signaling pathway. Upon infection, WRKY transcription factors are activated via phosphorylation by MAPKs and induce the expression of *VmAP2*/*ERF036* by promoter binding. However, putative downstream target genes *PDF1.2* and *PR* possessing GCC boxes in their promoters were not identified as interacting genes. It is possible that *VmAP2*/*ERF036* is not directly involved in activating *PR* genes, but may function by inducing the expression of other genes such as *VmAP2*/*ERF035* of this network, which may in turn lead to signal amplification and activation of expression of multiple pathogen resistance genes. It has been suggested that *VmAP2*/*ERF036* is a crucial component of MAPK cascades. Cell wall modifications are vital to plant pathogen resistance, and network analysis also revealed that *VmAP2*/*ERF036* may function together with cell wall alteration genes such as monooxygenase and β-glucosidase. Acyl-CoA-binding protein 4 (ACBP4) identified in the network, which participates in the biosynthesis of membrane lipids, had been shown to bind to RAP2.3 in the cytosol and at the periphery of the nucleus via an ankyrin domain [66]. It is also possible that interaction with *ACBP4* determines the stability, localization and transcriptional activity of *VmAP2*/*ERF036*. Diacylglycerol kinase (*DGK*), another hub gene of the transcriptome reported to be important for resistance to *Magnaporthe grisea* [67] and involved in other signal transduction mechanisms, was also detected in this network. The results indicate that *VmAP2*/*ERF036* may be the intersection point of a variety of signaling pathways. Overall, although the exact pathway and functional relevance of *VmAP2*/*ERF036* to pathogen resistance and its interactive genes remain to be determined, these data will serve as a valuable resource for candidate gene discovery and molecular mechanism elucidation related to pathogen response and signaling pathway regulation.

Comprehensive expression patterns analysis in various tissues indicates that, in addition to playing a vital role in fruit development and ripening, *VfAP2*/*ERF036* and *VmAP2*/*ERF036* are also involved in root defense and developmental processes. In addition, the orthologous genes *VfAP2*/*ERF036*/*VmAP2*/*ERF036* showed opposite expression patterns in *V. fordii* and *V. montana* after *F. oxysporum* inoculation. This finding indicates that this pair of orthologous genes may exert different functions via divergent signaling pathways and gene interactions. In view of the hub gene identity of *VmAP2*/*ERF036*, it is quite possible that *VmAP2*/*ERF036* is a putative key functional gene in response to *F. oxysporum*. Through genetic modification of *V. fordii* using the *VmAP2*/*ERF036* found in *V. montana*, it may be possible to enhance the disease-resistance capacity and productivity of the former.

In conclusion, a comprehensive investigation of a very important gene family, the AP2/ERF superfamily, was performed between susceptible *V*. *fordii* and resistant *V*. *montana*. This is the first comprehensive report on a transcriptome-wide survey and evolutionary analysis of AP2/ERF transcription factors in *Vernicia*. Our comparative analyses of the *Vf*/*VmAP2*/*ERF* superfamily will act as a first step toward disease-resistance breeding. Further comprehensive functional characterization of putative candidate *Vf/mAP2/ERF* genes identified in our study may contribute to revealing the mechanisms of resistance and controlling the *Fusarium* wilt disease, which will provide additional information for genetic engineering of *V. fordii* and possibly other beneficial plant species.

## Figures and Tables

**Table 1:** Summary of the classification and number of genes in the APETALA2/ethylene-responsive element binding factor (AP2/ERF) superfamily in *Vernicia montana*, *Vernicia fordii*, *Arabidopsis thaliana* and physic nut.

Family	Subfamily	Group	*Vernicia*		*Arabidopsis thaliana*	*Jatropha curcas*
			*V. montana*	*V. fordii*		
AP2		Total	14	12	18	16
ERF		Total	64	60	122	98
	DREB	Total	25	24	57	35
		I	5	6	10	5
		II	5	6	15	8
		III	13	10	23	16
		IV	2	2	9	6
	ERF	Total	39	36	65	63
		V	4	6	5	12
		VI	4	2	8	4
		VII	3	3	5	3
		VIII	6	5	15	12
		IX	15	12	17	21
		X	5	5	8	8
		VI-L	2	3	4	3
		Xb-L	0	0	3	0
RAV			2	2	6	4
Soloist			1	1	1	1
Total			81	75	147	119

The number of genes in the AP2/ERF family from *A. thaliana* and physic nut was reported by Nakano et al. [5] and Tang et al. [25]. DREB: dehydration-responsive element-binding; RAV: related to abscisic acid insensitive 3 (ABI3)/ VP1.

**Table 2:** Role of ERFs in response to fungal pathogens encountered by plants.

Candidate Gene	Group	Regulation	Source Plant	Fungal Pathogen	References
*ERF1*	IX	activator	*A. thaliana*	*F. oxysporum*	[29]
*AtERF2*	IX	activator	*A. thaliana*	*F. oxysporum*	[1]
*AtERF4*	VIII	repressor	*A. thaliana*	*F. oxysporum*	[1]
*AtERF14*	IX	activator	*A. thaliana*	*F. oxysporum*	[31]
*ERF5*	IX	activator	*A. thaliana*	*Botrytis cinerea*	[32]
*ERF6*	IX	activator	*A. thaliana*	*B. cinerea*	[2,32]
*ERF9*	VIII	repressor	*A. thaliana*	*B. cinerea*	[60]
*AtERF15*	IX	activator	*A. thaliana*	*B. cinerea*	[34]
*ORA59*	IX	activator	*A. thaliana*	*B. cinerea*	[35]
*VpERF3*	IX	activator	*Vitis pseudoreticulata*	*Phytophtora parasitica*	[61]
*OsERF922*	IX	repressor	*Oryza sativa*	*Magnaporthe oryzae*	[62]
*TaPIE1*	IX	activator	*Triticum aestivum*	*Rhizoctonia cerealis*	[63]
*GmERF3*	IV	activator	*Glycine max*	*Alternaria alternate*	[18]
*OPBP1*	IX	activator	*Nicotiana tabacum*	*Rhizoctonia solani*	[64]

## Supplementary Materials

Supplementary materials can be found at http://www.mdpi.com/2073-4425/7/12/109/s1. File S1: Phylogenetic analysis of AP2/ERF superfamily proteins between *V. fordii*, *V. montana* and *Arabidopsis thaliana*, Figure S1: Alignment of APETALA2/ethylene-responsive element binding factor (AP2/ERF) domains of 124 ERF family proteins identified from *Vernicia fordii* and *Vernicia montana*, Figure S2: Alignment of AP2/ERF domains of 26 AP2 family proteins identified from *V. fordii* and *V. montana*, Figure S3: Frequency of ERFs in response to *Fusarium oxysporum* in phylogenetic groups, Figure S4: Comparative analysis of the expression trends of 53 pairs of orthologous genes in response to *F. oxysporum*, Figure S5: Comparative analysis of the expression levels of the selected *AP2/ERF* genes in root tissue, Figure S6: Expression trends of AP2/ERF genes for tissue specific expression analysis in *Vernicia* during the four infection stages, Figure S7: Clusters of orthologous groups (COG) classification, Figure S8: Expression profiles of *AP2* genes in response to *F. oxysporum* in the root of *V. fordii* and *V. montana*, Table S1: The individual APETALA2/ethylene-responsive element binding factor (AP2/ERF) genes identified in *Vernicia fordii* and *Vernicia montana* transcriptome database, Table S2: Conserved motifs identified from the ERF family in *V. fordii* and *V. montana*, Table S3: The one to one orthologous gene pairs and their corresponding Ka/Ks ratios, Table S4: List of specific primer sequences used for real-time PCR analysis, Table S5: Pearson correlation *r* between transcriptome data and qRT-PCR result of selected orthologous genes in *V. fordii* and *V. montana.*
