# Supplementary material for: Divergent Expression Patterns in Two Vernicia Species Revealed the Potential Role of the Hub Gene VmAP2/ERF036 in Resistance to Fusarium oxysporum in Vernicia montana
Source: Genes (Basel). 2016 Dec 1;7(12):109. doi: 10.3390/genes7120109 (PMC5192485; doi:10.3390/genes7120109)
Supplement: Supplementary file 1 [file genes-07-00109-s001.zip › genes-152598-supplementary-final.pdf]

Supplementary Materials: Divergent Expression Patterns in Two *Vernicia* Species Revealed the Potential Role of the Hub Gene *VmAP2/ERF036* in Resistance to *Fusarium oxysporum* in *Vernicia montana*

Qiyan Zhang, Ming Gao, Liwen Wu, Yangdong Wang and Yicun Chen

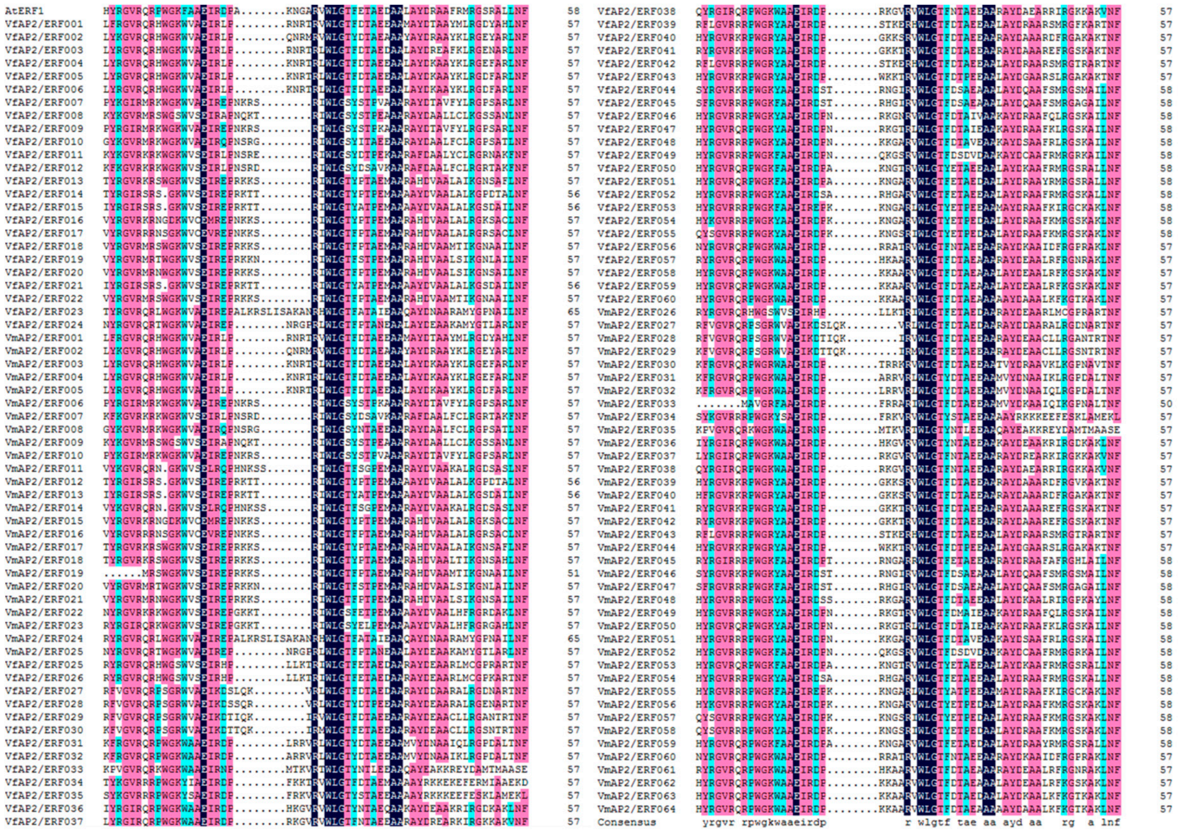

Figure S1. Alignment of APETALA2/ethylene-responsive element binding factor (AP2/ERF) domains of 124 ERF family proteins identified from *Vernicia fordii* and *Vernicia montana*. Multiple sequence alignment was conducted with BioEdit and DNAMAN. The GCC box-binding domain of *Arabidopsis* AtERF1 (At4g17500) is indicated at the top. **Blue shadow**: conserved amino acid residues (>50%); **Pink shadow**: conserved amino acid residues (>75%); **Black shadow**: 100% conserved amino acid residues.

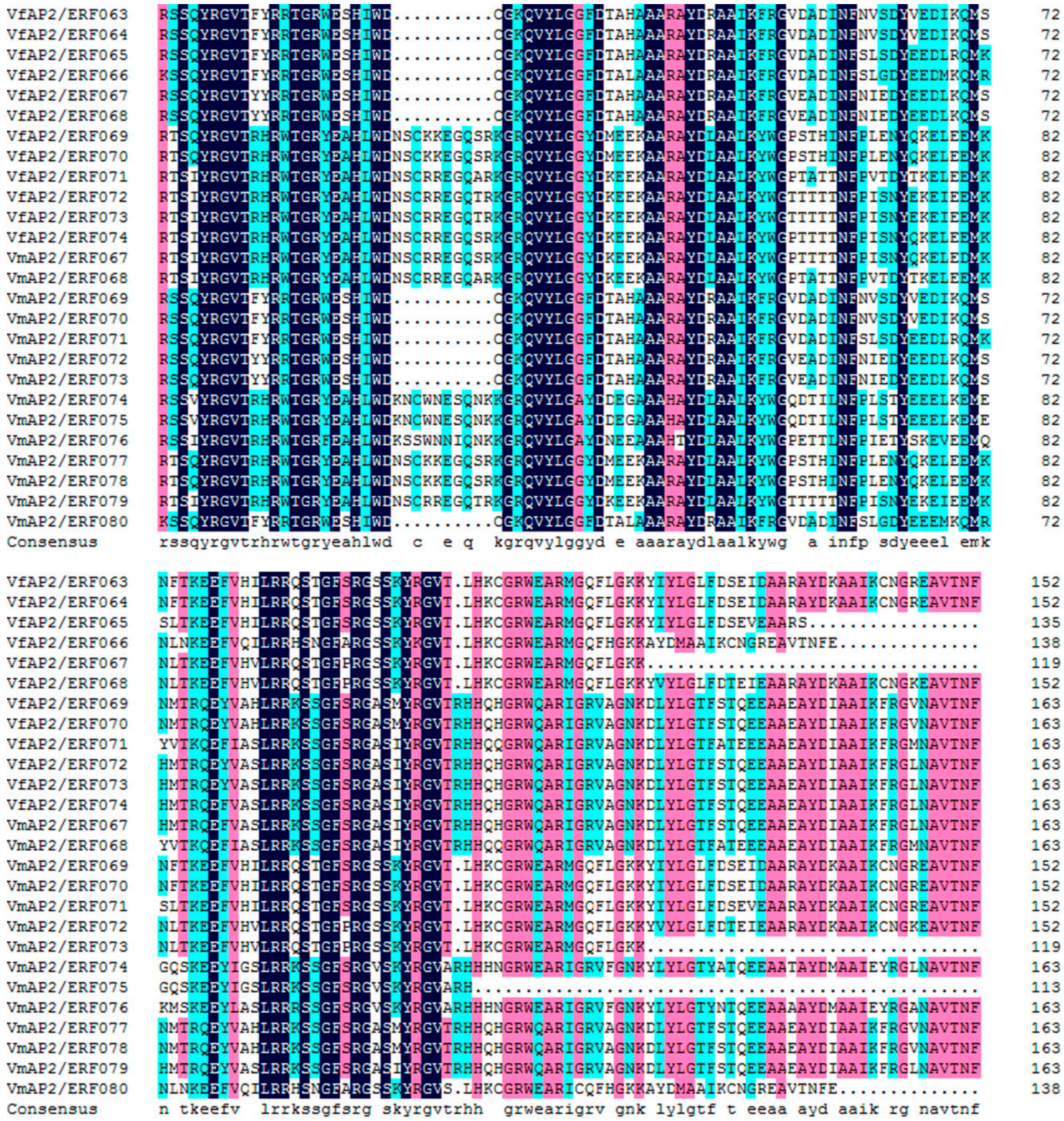

Figure S2. Alignment of AP2/ERF domains of 26 AP2 family proteins identified from *V. fordii* and *V. montana*. Multiple sequence alignment was conducted with BioEdit and DNAMAN. **Blue shadow**: conserved amino acid residues (>50%); **Pink shadow**: conserved amino acid residues (>75%); **Black shadow**: 100% conserved amino acid residues.

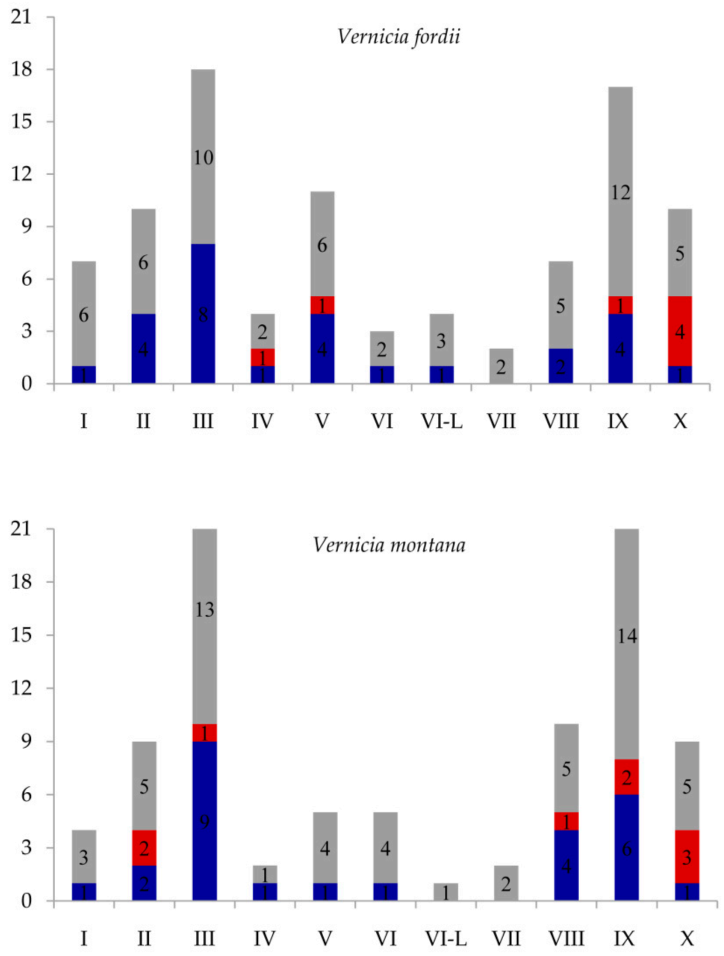

Figure S3. Frequency of ERFs in response to *Fusarium oxysporum* in phylogenetic groups. The full bar indicates the number of ERFs with more than twofold change in at least one infected experiment per group. **Red** indicates members with more than twofold upregulated expression at all the three infected periods. **Blue** indicates members with more than twofold downregulated.

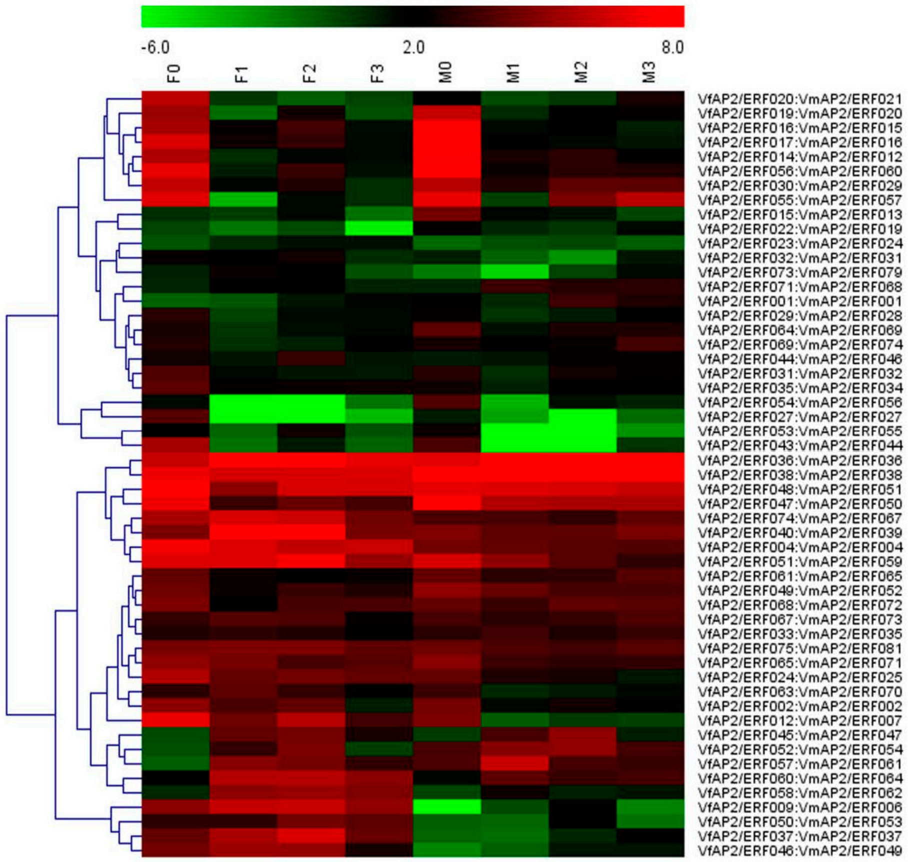

Figure S4. Comparative analysis of the expression trends of 53 pairs of orthologous genes in response to *F. oxysporum*. The heat-map has been generated based on the log2 transformed reads per kilobase per million mapped reads (RPKM) values and the color scale is shown at the top. **Red** represents high expression and **green** represents low expression.

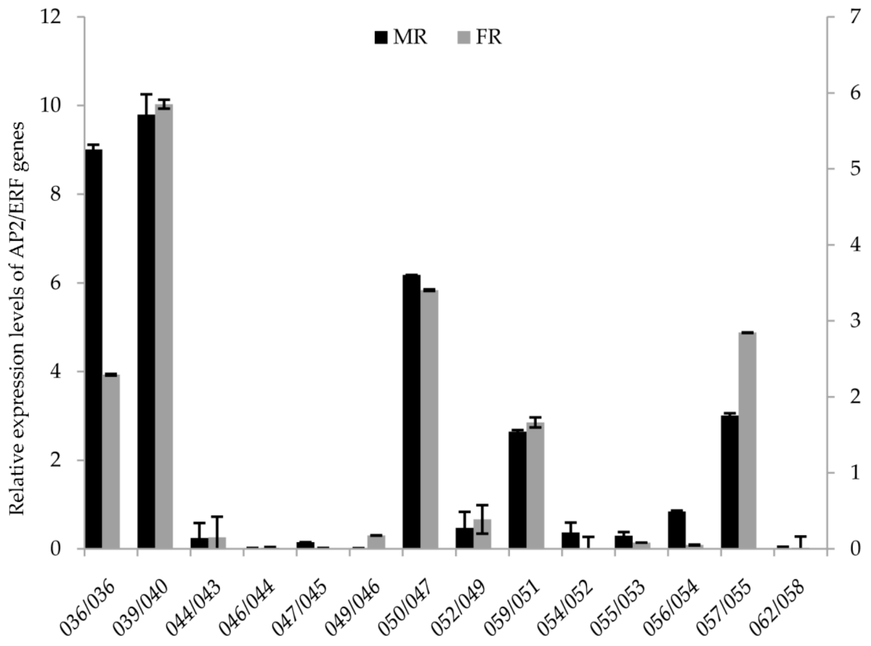

Figure S5. Comparative analysis of the expression levels of the selected *AP2/ERF* genes in root tissue. MR: root tissue in *V. montana;* FR: root tissue in *V. fordii*.

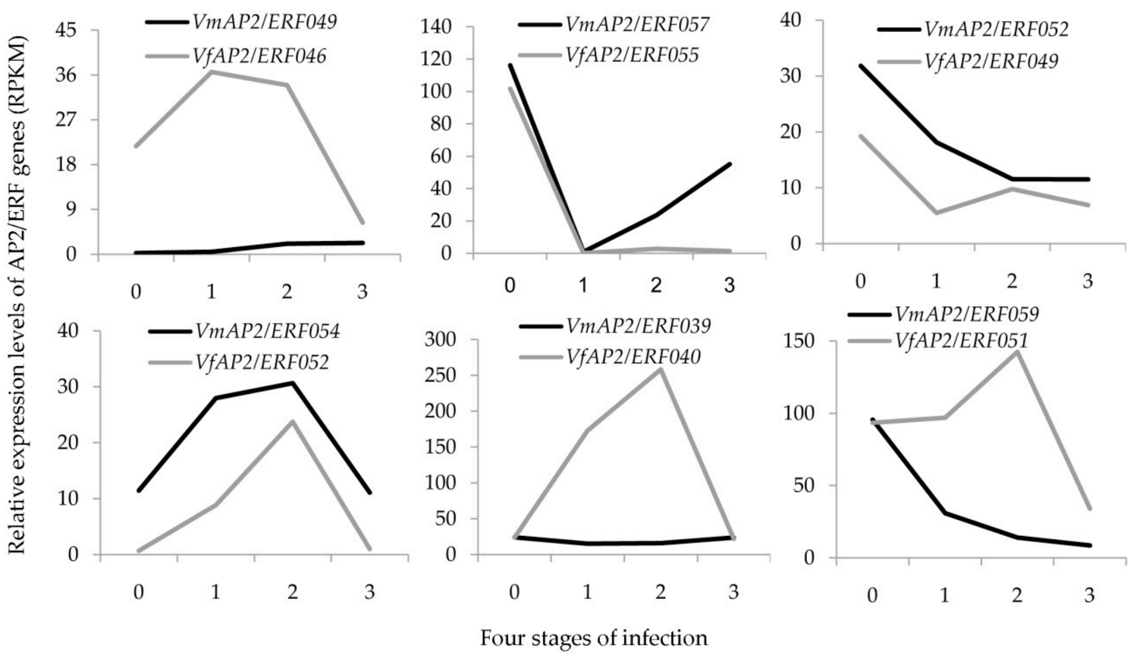

Figure S6. Expression trends of AP2/ERF genes for tissue specific expression analysis in *Vernicia* during the four infection stages. Axis represents averaged RPKM values from three biological experiments. The numbers in the *x*-axis represent stage of infection, as follows: 0, uninfected stage; 1, early stage of infection; 2, middle stage of infection; 3, late stage of infection.

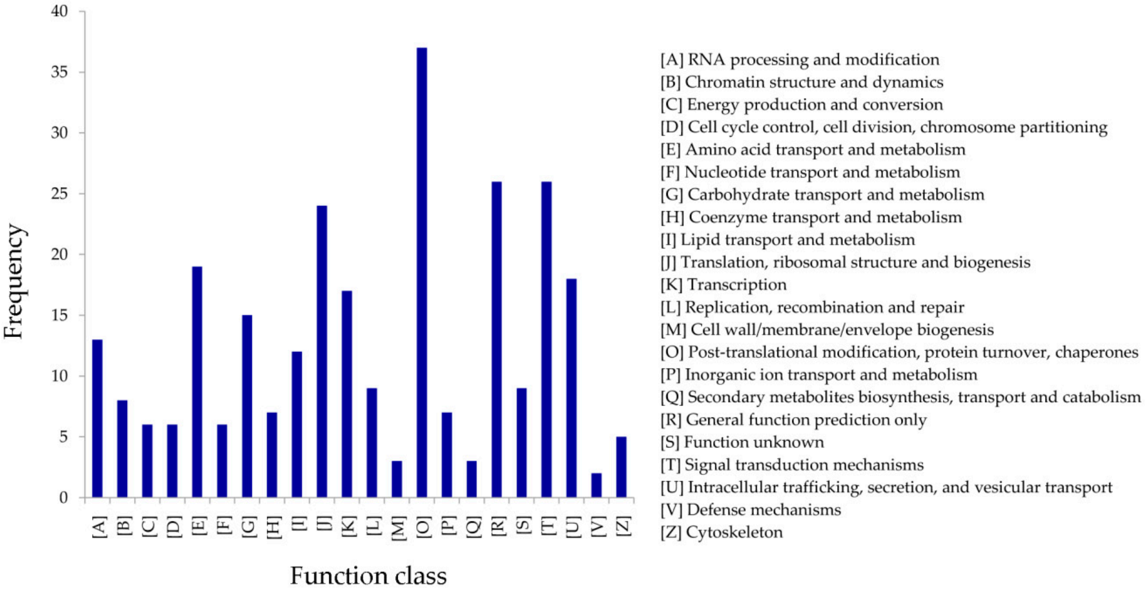

Figure S7. Clusters of orthologous groups (COG) classification. In total, 278 functional genes of whole annotated unigenes were grouped into 22 classifications.

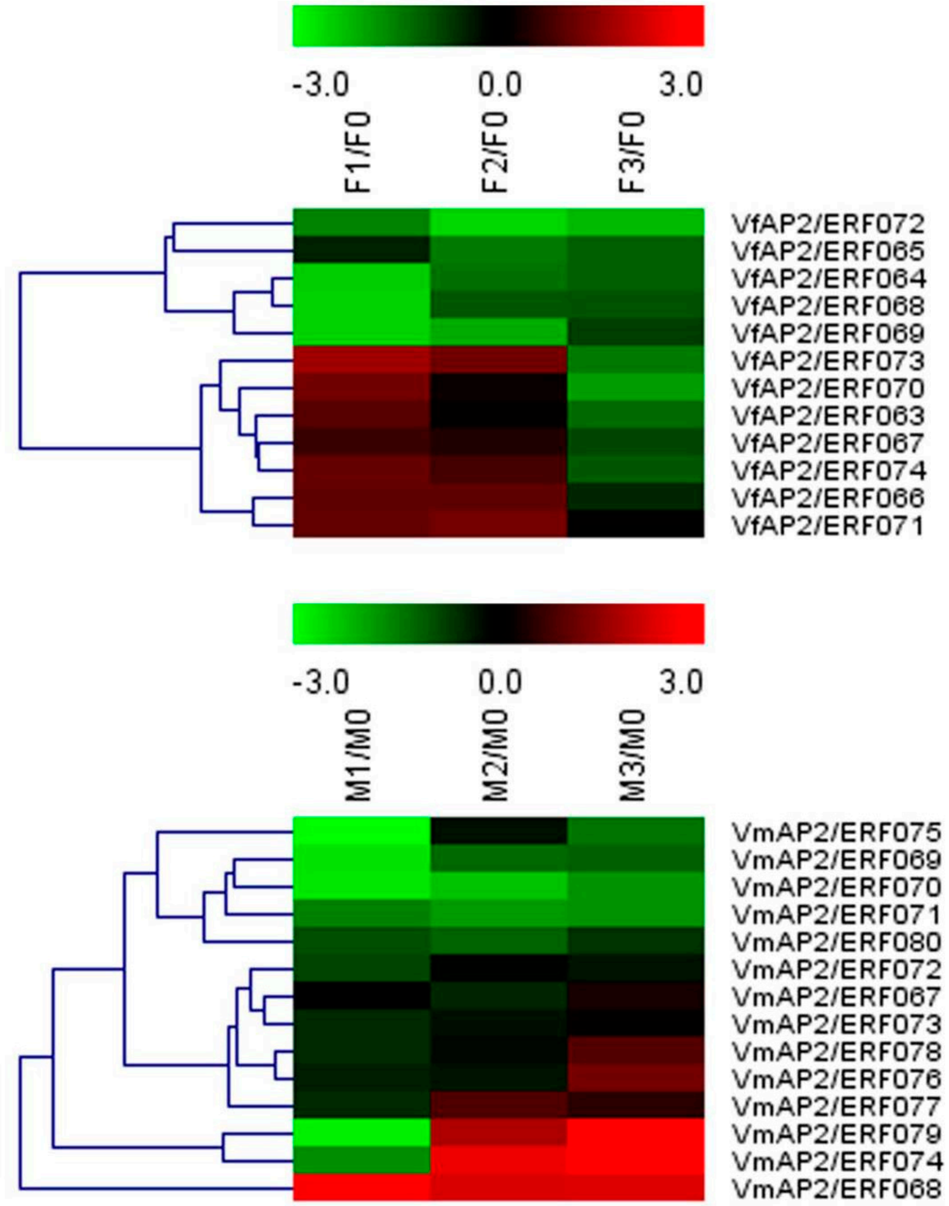

Figure S8. Expression profiles of *AP2* genes in response to *F. oxysporum* in the root of *V. fordii* and *V. montana*. The heatmap has been generated based on the log2-transformed fold-change RPKM values and the color scale is shown at the top. **Red** represents upregulation and **green** represents downregulation.

Table S1. The individual APETALA2/ethylene-responsive element binding factor (*AP2*/*ERF*) genes identified in *V. fordii* and *V. montana* transcriptome database.

Group	Transcript_ID	GeneticName	Accession No.	Effective_Length	Annotation
Group1	comp183962_c0_seq1	*VfAP2/ERF001*	KX868880	1420.00	EREBP-like factor
Group1	comp189672_c0_seq2	*VfAP2/ERF002*	KX868881	1823.46	EREBP-like factor
Group1	comp185524_c0_seq1	*VfAP2/ERF003*	KX868882	1915.00	EREBP-like factor
Group1	comp160673_c0_seq2	*VfAP2/ERF004*	KX868883	1150.82	EREBP-like factor
Group1	comp190709_c2_seq5	*VfAP2/ERF005*	KX868884	1538.54	EREBP-like factor
Group1	comp192642_c0_seq1	*VfAP2/ERF006*	KX868885	2520.00	EREBP-like factor
Group2	comp84118_c0_seq1	*VfAP2/ERF007*	KX868886	462.00	EREBP-like factor
Group2	comp149201_c0_seq1	*VfAP2/ERF008*	KX868887	459.00	EREBP-like factor
Group2	comp176401_c0_seq4	*VfAP2/ERF009*	KX868888	1411.21	EREBP-like factor

Group2	comp160987_c0_seq1	*VfAP2/ERF010*	KX868889	602.00	EREBP-like factor
Group2	comp160830_c0_seq2	*VfAP2/ERF011*	KX868890	563.42	EREBP-like factor
Group2	comp184966_c0_seq2	*VfAP2/ERF012*	KX868891	1048.51	EREBP-like factor
Group3	comp159254_c1_seq1	*VfAP2/ERF013*	KX868892	405.00	EREBP-like factor
Group3	comp180080_c0_seq3	*VfAP2/ERF014*	KX868893	1570.07	EREBP-like factor
Group3	comp169343_c0_seq2	*VfAP2/ERF015*	KX868894	1213.62	EREBP-like factor
Group3	comp181674_c0_seq1	*VfAP2/ERF016*	KX868895	972.04	EREBP-like factor
Group3	comp181389_c0_seq1	*VfAP2/ERF017*	KX868896	1182.00	EREBP-like factor
Group3	comp173409_c0_seq1	*VfAP2/ERF018*	KX868897	824.84	EREBP-like factor
Group3	comp183897_c0_seq3	*VfAP2/ERF019*	KX868898	1046.09	EREBP-like factor
Group3	comp176265_c0_seq1	*VfAP2/ERF020*	KX868899	879.01	EREBP-like factor
Group3	comp164645_c0_seq1	*VfAP2/ERF021*	KX868900	661.00	EREBP-like factor
Group3	comp173281_c0_seq1	*VfAP2/ERF022*	KX868901	804.00	EREBP-like factor
Group4	comp171489_c1_seq1	*VfAP2/ERF023*	KX868902	714.00	EREBP-like factor
Group4	comp189934_c0_seq4	*VfAP2/ERF024*	KX868903	850.79	EREBP-like factor
Group5	comp148732_c0_seq1	*VfAP2/ERF025*	KX868904	432.00	EREBP-like factor
Group5	comp166681_c0_seq2	*VfAP2/ERF026*	KX868905	726.43	EREBP-like factor
Group5	comp158438_c0_seq3	*VfAP2/ERF027*	KX868906	1528.82	EREBP-like factor
Group5	comp149900_c0_seq1	*VfAP2/ERF028*	KX868907	1294.00	EREBP-like factor
Group5	comp181196_c0_seq1	*VfAP2/ERF029*	KX868908	1095.15	EREBP-like factor
Group5	comp188688_c0_seq1	*VfAP2/ERF030*	KX868909	1229.26	EREBP-like factor
Group6	comp189868_c0_seq1	*VfAP2/ERF031*	KX868910	1820.54	EREBP-like factor
Group6	comp186790_c1_seq1	*VfAP2/ERF032*	KX868911	1526.84	EREBP-like factor
Group6-l	comp192264_c0_seq1	*VfAP2/ERF033*	KX868912	1971.00	EREBP-like factor
Group6-l	comp116494_c0_seq1	*VfAP2/ERF034*	KX868913	864.00	EREBP-like factor
Group6-l	comp183367_c0_seq5	*VfAP2/ERF035*	KX868914	1480.92	EREBP-like factor
Group7	comp178925_c0_seq2	*VfAP2/ERF036*	KX868915	747.64	EREBP-like factor
Group7	comp180742_c0_seq2	*VfAP2/ERF037*	KX868916	1335.90	EREBP-like factor
Group7	comp187442_c0_seq1	*VfAP2/ERF038*	KX868917	1701.00	EREBP-like factor
Group8	comp101758_c0_seq1	*VfAP2/ERF039*	KX868918	336.00	EREBP-like factor
Group8	comp179927_c0_seq1	*VfAP2/ERF040*	KX868919	1018.00	EREBP-like factor
Group8	comp177967_c0_seq1	*VfAP2/ERF041*	KX868920	742.00	EREBP-like factor
Group8	comp157807_c0_seq2	*VfAP2/ERF042*	KX868921	956.08	EREBP-like factor
Group8	comp160872_c0_seq1	*VfAP2/ERF043*	KX868922	806.00	EREBP-like factor
Group9	comp172871_c0_seq1	*VfAP2/ERF044*	KX868923	612.00	EREBP-like factor
Group9	comp174782_c0_seq3	*VfAP2/ERF045*	KX868924	864.06	EREBP-like factor
Group9	comp175038_c0_seq1	*VfAP2/ERF046*	KX868925	667.00	EREBP-like factor
Group9	comp189715_c1_seq1	*VfAP2/ERF047*	KX868926	964.00	EREBP-like factor
Group9	comp192585_c1_seq1	*VfAP2/ERF048*	KX868927	1237.00	EREBP-like factor
Group9	comp179276_c0_seq1	*VfAP2/ERF049*	KX868928	1501.09	EREBP-like factor
Group9	comp186047_c0_seq1	*VfAP2/ERF050*	KX868929	531.42	EREBP-like factor
Group9	comp181356_c0_seq5	*VfAP2/ERF051*	KX868930	792.80	EREBP-like factor
Group9	comp171620_c0_seq1	*VfAP2/ERF052*	KX868931	825.00	EREBP-like factor
Group9	comp181105_c0_seq2	*VfAP2/ERF053*	KX868932	843.07	EREBP-like factor
Group9	comp136851_c0_seq1	*VfAP2/ERF054*	KX868933	768.00	EREBP-like factor
Group9	comp178204_c0_seq1	*VfAP2/ERF055*	KX868934	952.00	EREBP-like factor

Group10	comp174721_c0_seq1	*VfAP2/ERF056*	KX868935	1173.00	EREBP-like factor
Group10	comp184569_c0_seq5	*VfAP2/ERF057*	KX868936	1684.12	EREBP-like factor
Group10	comp181627_c0_seq2	*VfAP2/ERF058*	KX868937	1352.93	EREBP-like factor
Group10	comp181660_c0_seq2	*VfAP2/ERF059*	KX868938	1323.16	EREBP-like factor
Group10	comp194219_c2_seq5	*VfAP2/ERF060*	KX868939	1147.02	EREBP-like factor
RAV	comp189189_c0_seq2	*VfAP2/ERF061*	KX868940	1061.98	RAV-like factor
RAV	comp176028_c0_seq1	*VfAP2/ERF062*	KX868941	1300.00	RAV-like factor
AP2	comp192732_c0_seq6	*VfAP2/ERF063*	KX868942	796.07	AP2-like factor, euAP2 lineage
AP2	comp191512_c0_seq2	*VfAP2/ERF064*	KX868943	1193.76	AP2-like factor, euAP2 lineage
AP2	comp195632_c1_seq20	*VfAP2/ERF065*	KX868944	1373.64	AP2-like factor, euAP2 lineage
AP2	comp191268_c0_seq4	*VfAP2/ERF066*	KX868945	1482.02	AP2-like factor, euAP2 lineage
AP2	comp184116_c0_seq1	*VfAP2/ERF067*	KX868946	1035.46	AP2-like factor, euAP2 lineage
AP2	comp186054_c0_seq1	*VfAP2/ERF068*	KX868947	1949.28	AP2-like factor, euAP2 lineage
AP2	comp179411_c0_seq1	*VfAP2/ERF069*	KX868948	1795.38	AP2-like factor, ANT lineage
AP2	comp178775_c0_seq3	*VfAP2/ERF070*	KX868949	1188.85	AP2-like factor, ANT lineage
AP2	comp191827_c0_seq1	*VfAP2/ERF071*	KX868950	2271.00	AP2-like factor, ANT lineage
AP2	comp184446_c1_seq1	*VfAP2/ERF072*	KX868951	1584.00	AP2-like factor, ANT lineage
AP2	comp184446_c0_seq1	*VfAP2/ERF073*	KX868952	732.00	AP2-like factor, ANT lineage
AP2	comp184881_c0_seq5	*VfAP2/ERF074*	KX868953	1055.28	AP2-like factor, ANT lineage
solosist	comp191237_c1_seq13	*VfAP2/ERF075*	KX868954	1272.47	-
Group1	comp138429_c0_seq4	*VmAP2/ERF001*	KX868799	1510.61	EREBP-like factor
Group1	comp164853_c0_seq3	*VmAP2/ERF002*	KX868800	2120.15	EREBP-like factor
Group1	comp137524_c0_seq1	*VmAP2/ERF003*	KX868801	2219.00	EREBP-like factor
Group1	comp64502_c0_seq1	*VmAP2/ERF004*	KX868802	1676.00	EREBP-like factor
Group1	comp164758_c0_seq1	*VmAP2/ERF005*	KX868803	1245	EREBP-like factor
Group2	comp116109_c0_seq1	*VmAP2/ERF006*	KX868804	1132.00	EREBP-like factor
Group2	comp157844_c0_seq1	*VmAP2/ERF007*	KX868805	1068.00	EREBP-like factor
Group2	comp128472_c0_seq1	*VmAP2/ERF008*	KX868806	580.33	EREBP-like factor
Group2	comp129700_c0_seq1	*VmAP2/ERF009*	KX868807	418.00	EREBP-like factor
Group2	comp186932_c0_seq1	*VmAP2/ERF010*	KX868808	466.00	EREBP-like factor
Group3	comp251319_c0_seq1	*VmAP2/ERF011*	KX868809	431.00	EREBP-like factor
Group3	comp124868_c0_seq1	*VmAP2/ERF012*	KX868810	1177.00	EREBP-like factor
Group3	comp128319_c0_seq1	*VmAP2/ERF013*	KX868811	774.65	EREBP-like factor
Group3	comp143146_c0_seq1	*VmAP2/ERF014*	KX868812	1004	EREBP-like factor
Group3	comp138574_c0_seq4	*VmAP2/ERF015*	KX868813	1122.79	EREBP-like factor
Group3	comp129405_c0_seq1	*VmAP2/ERF016*	KX868814	1216.00	EREBP-like factor
Group3	comp85604_c0_seq1	*VmAP2/ERF017*	KX868815	548.00	EREBP-like factor
Group3	comp107282_c0_seq1	*VmAP2/ERF018*	KX868816	728.14	EREBP-like factor
Group3	comp143204_c0_seq1	*VmAP2/ERF019*	KX868817	917.00	EREBP-like factor
Group3	comp127938_c0_seq1	*VmAP2/ERF020*	KX868818	1056.00	EREBP-like factor
Group3	comp145334_c1_seq1	*VmAP2/ERF021*	KX868819	928.31	EREBP-like factor

Group3	comp151923_c1_seq1	*VmAP2/ERF022*	KX868820	545.50	EREBP-like factor
Group3	comp208000_c0_seq1	*VmAP2/ERF023*	KX868821	882.00	EREBP-like factor
Group4	comp397454_c0_seq1	*VmAP2/ERF024*	KX868822	706.00	EREBP-like factor
Group4	comp144341_c0_seq1	*VmAP2/ERF025*	KX868823	1078.00	EREBP-like factor
Group5	comp63102_c0_seq1	*VmAP2/ERF026*	KX868824	506.00	EREBP-like factor
Group5	comp110005_c0_seq1	*VmAP2/ERF027*	KX868825	1074.00	EREBP-like factor
Group5	comp150298_c0_seq3	*VmAP2/ERF028*	KX868826	795.88	EREBP-like factor
Group5	comp157176_c0_seq1	*VmAP2/ERF029*	KX868827	1026.69	EREBP-like factor
Group6	comp129421_c0_seq1	*VmAP2/ERF030*	KX868828	1099.00	EREBP-like factor
Group6	comp137242_c0_seq2	*VmAP2/ERF031*	KX868829	1513.65	EREBP-like factor
Group6	comp137462_c0_seq3	*VmAP2/ERF032*	KX868830	1707.28	EREBP-like factor
Group6	comp161719_c1_seq4	*VmAP2/ERF033*	KX868831	867.29	EREBP-like factor
Group6-l	comp160313_c0_seq1	*VmAP2/ERF034*	KX868832	1412.00	EREBP-like factor
Group6-l	comp162707_c0_seq1	*VmAP2/ERF035*	KX868833	2013.00	EREBP-like factor
Group7	comp139704_c0_seq1	*VmAP2/ERF036*	KX868834	2079.00	EREBP-like factor
Group7	comp151256_c0_seq3	*VmAP2/ERF037*	KX868835	997.94	EREBP-like factor
Group7	comp162529_c0_seq1	*VmAP2/ERF038*	KX868836	1536.01	EREBP-like factor
Group8	comp146876_c0_seq1	*VmAP2/ERF039*	KX868837	1089.00	EREBP-like factor
Group8	comp149810_c0_seq1	*VmAP2/ERF040*	KX868838	1518.34	EREBP-like factor
Group8	comp128719_c0_seq1	*VmAP2/ERF041*	KX868839	1098.00	EREBP-like factor
Group8	comp140154_c0_seq1	*VmAP2/ERF042*	KX868840	1365.00	EREBP-like factor
Group8	comp141925_c0_seq1	*VmAP2/ERF043*	KX868841	341.75	EREBP-like factor
Group8	comp173049_c0_seq1	*VmAP2/ERF044*	KX868842	795.00	EREBP-like factor
Group9	comp227613_c0_seq1	*VmAP2/ERF045*	KX868843	420.00	EREBP-like factor
Group9	comp139267_c0_seq1	*VmAP2/ERF046*	KX868844	870.00	EREBP-like factor
Group9	comp144276_c0_seq3	*VmAP2/ERF047*	KX868845	1155.52	EREBP-like factor
Group9	comp109538_c0_seq1	*VmAP2/ERF048*	KX868846	650.00	EREBP-like factor
Group9	comp163543_c0_seq2	*VmAP2/ERF049*	KX868847	1056.75	EREBP-like factor
Group9	comp141156_c0_seq3	*VmAP2/ERF050*	KX868848	1144.45	EREBP-like factor
Group9	comp155356_c1_seq1	*VmAP2/ERF051*	KX868849	1230.00	EREBP-like factor
Group9	comp145295_c0_seq1	*VmAP2/ERF052*	KX868850	1078.00	EREBP-like factor
Group9	comp149797_c0_seq1	*VmAP2/ERF053*	KX868851	1037.00	EREBP-like factor
Group9	comp129078_c0_seq3	*VmAP2/ERF054*	KX868852	1034.51	EREBP-like factor
Group9	comp137888_c0_seq1	*VmAP2/ERF055*	KX868853	919.00	EREBP-like factor
Group9	comp79448_c0_seq1	*VmAP2/ERF056*	KX868854	766.00	EREBP-like factor
Group9	comp149261_c0_seq1	*VmAP2/ERF057*	KX868855	845.31	EREBP-like factor
Group9	comp156406_c1_seq2	*VmAP2/ERF058*	KX868856	379.16	EREBP-like factor
Group9	comp128686_c0_seq1	*VmAP2/ERF059*	KX868857	1229.00	EREBP-like factor
Group10	comp122372_c0_seq1	*VmAP2/ERF060*	KX868858	1976.49	EREBP-like factor
Group10	comp154312_c0_seq2	*VmAP2/ERF061*	KX868859	1766.91	EREBP-like factor
Group10	comp154195_c0_seq1	*VmAP2/ERF062*	KX868860	1201.03	EREBP-like factor
Group10	comp143779_c0_seq1	*VmAP2/ERF063*	KX868861	795.00	EREBP-like factor
Group10	comp162260_c0_seq3	*VmAP2/ERF064*	KX868862	720.48	EREBP-like factor
RAV	comp159597_c0_seq2	*VmAP2/ERF065*	KX868863	1253.54	RAV-like factor
RAV	comp169832_c0_seq1	*VmAP2/ERF066*	KX868864	1305.00	RAV-like factor

AP2	comp159240_c0_seq11	*VmAP2/ERF067*	KX868865	1080.48	AP2-like factor, ANT lineage
AP2	comp165608_c0_seq13	*VmAP2/ERF068*	KX868866	2246.56	AP2-like factor, ANT lineage
AP2	comp166307_c0_seq9	*VmAP2/ERF069*	KX868867	1326.76	AP2-like factor, euAP2 lineage
AP2	comp166849_c0_seq3	*VmAP2/ERF070*	KX868868	1041.65	AP2-like factor, euAP2 lineage
AP2	comp165181_c0_seq84	*VmAP2/ERF071*	KX868869	2623.48	AP2-like factor, euAP2 lineage
AP2	comp156517_c1_seq1	*VmAP2/ERF072*	KX868870	1899.00	AP2-like factor, euAP2 lineage
AP2	comp165500_c1_seq5	*VmAP2/ERF073*	KX868871	1306.33	AP2-like factor, euAP2 lineage
AP2	comp105036_c0_seq1	*VmAP2/ERF074*	KX868872	942.00	AP2-like factor, ANT lineage
AP2	comp86450_c0_seq1	*VmAP2/ERF075*	KX868873	561.00	AP2-like factor, ANT lineage
AP2	comp293964_c0_seq1	*VmAP2/ERF076*	KX868874	1269.00	AP2-like factor, ANT lineage
AP2	comp111892_c0_seq2	*VmAP2/ERF077*	KX868875	1085.13	AP2-like factor, ANT lineage
AP2	comp149426_c0_seq2	*VmAP2/ERF078*	KX868876	1275.65	AP2-like factor, ANT lineage
AP2	comp104878_c0_seq1	*VmAP2/ERF079*	KX868877	1493.00	AP2-like factor, ANT lineage
AP2	comp155189_c0_seq1	*VmAP2/ERF080*	KX868878	1066.98	AP2-like factor, euAP2 lineage
solosist	comp167675_c0_seq10	*VmAP2/ERF081*	KX868879	1694.73	-

Table S2. Conserved motifs identified from the ERF family in *V. fordii* and *V. montana.*

MOTIF-1	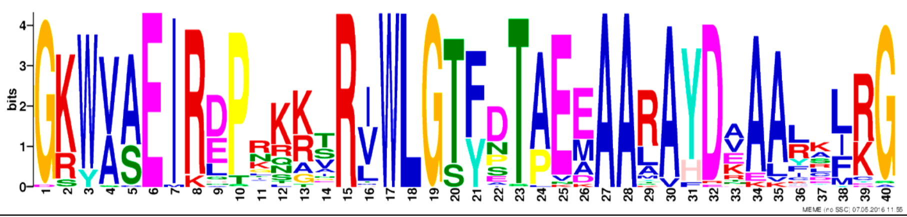
MOTIF-2	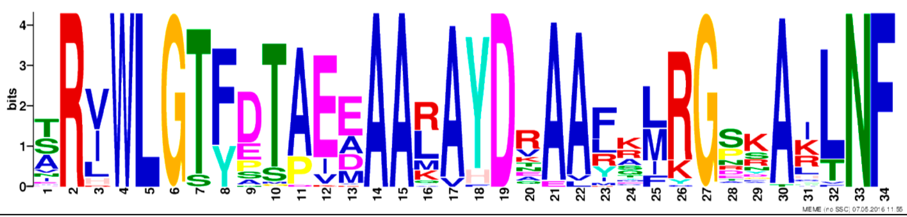
MOTIF-3	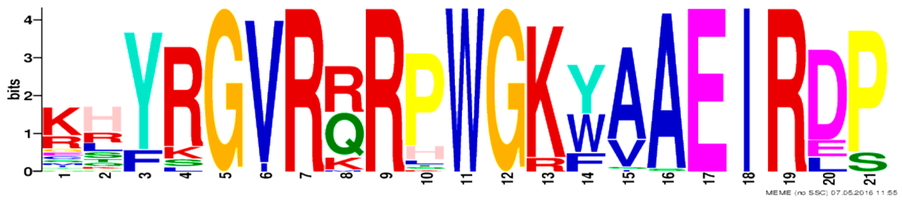

MOTIF-4	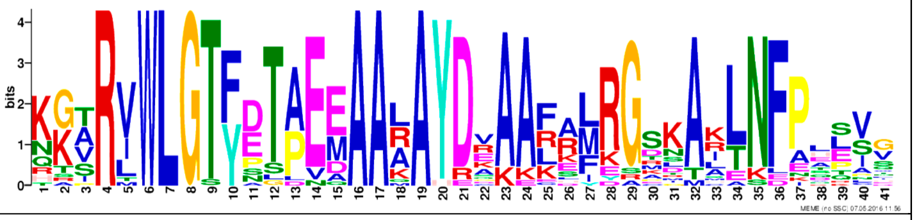
MOTIF-5	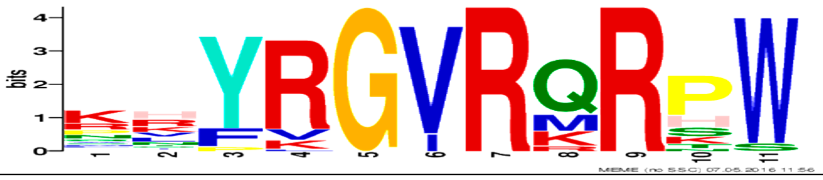
MOTIF-6	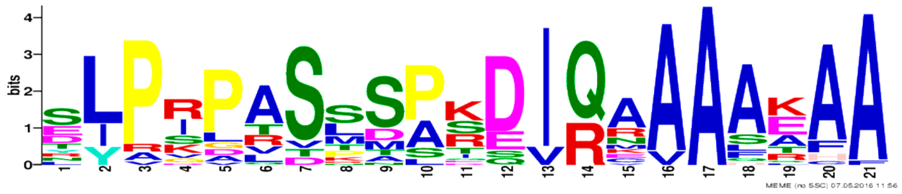
MOTIF-7	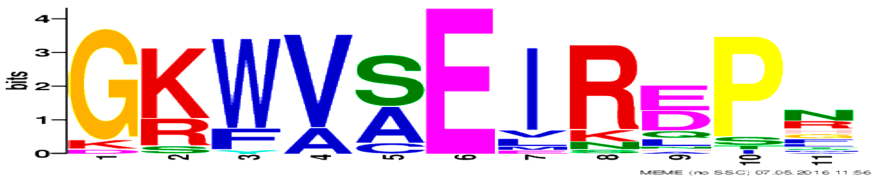
MOTIF-8	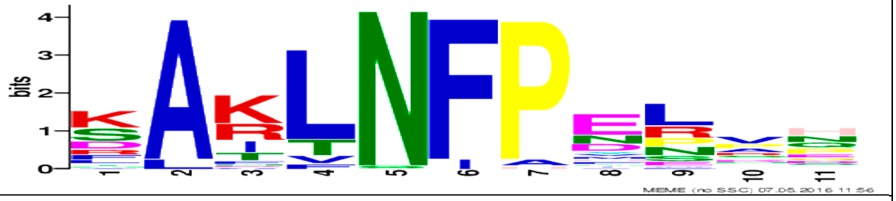
MOTIF-9	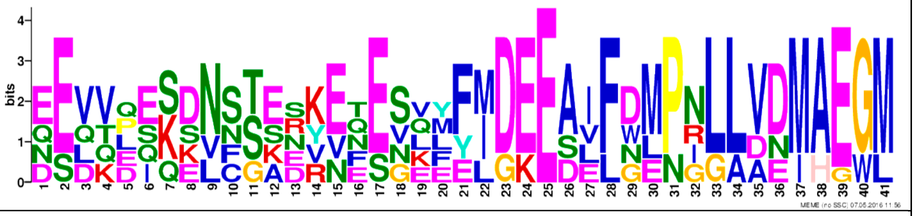
MOTIF-10	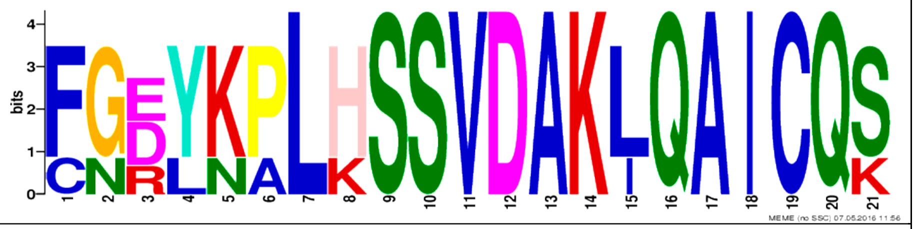
MOTIF-11	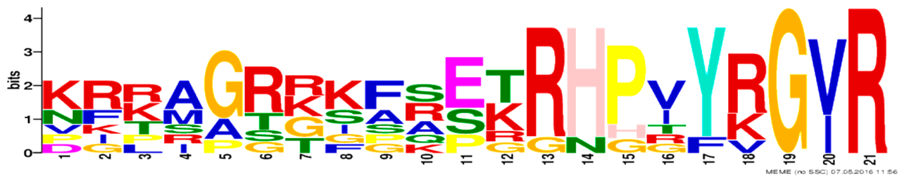

MOTIF-12	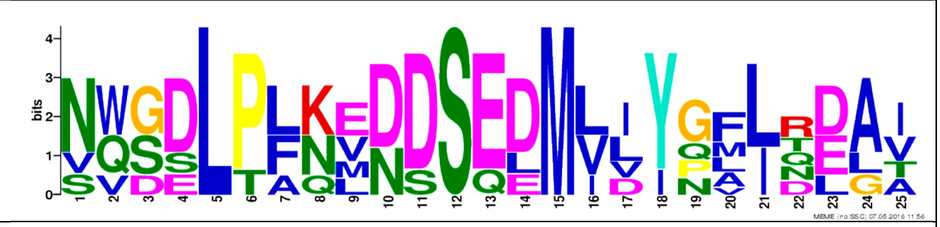
MOTIF-13	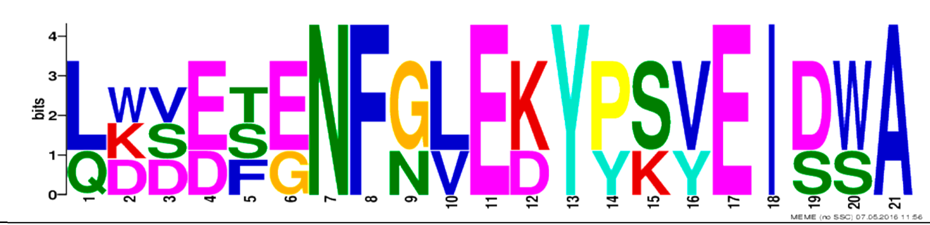
MOTIF-14	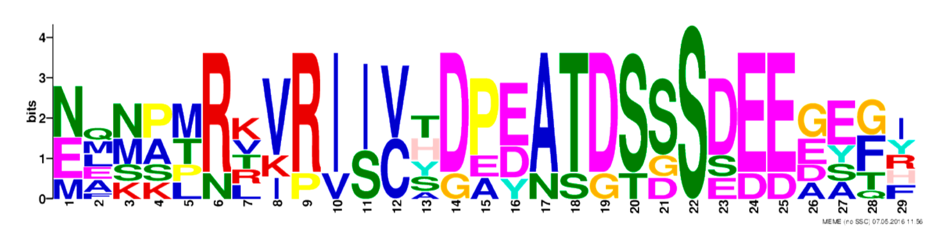
MOTIF-15	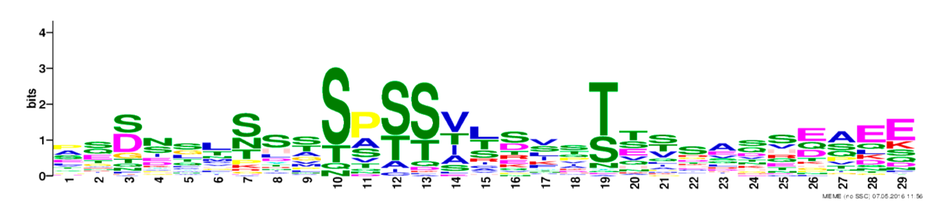
MOTIF-16	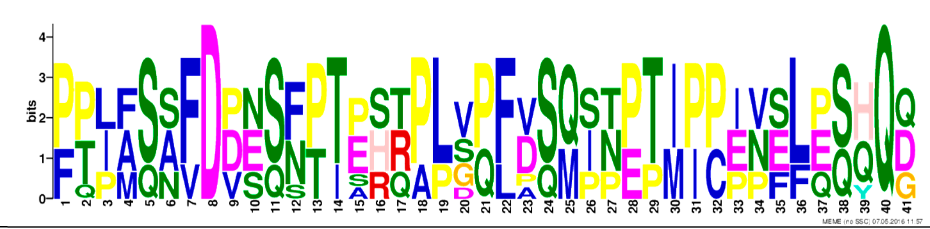
MOTIF-17	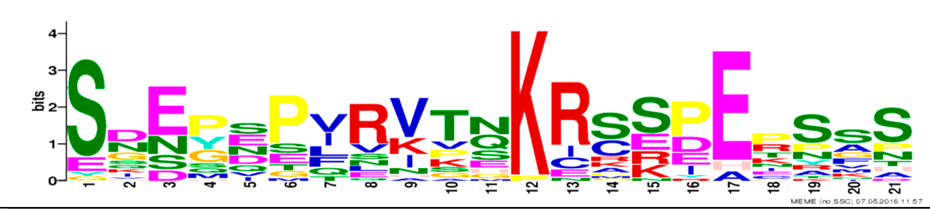
MOTIF-18	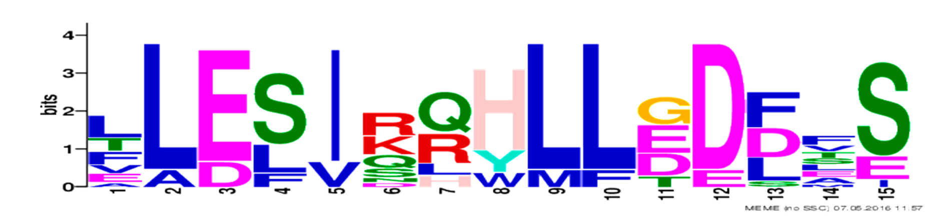
MOTIF-19	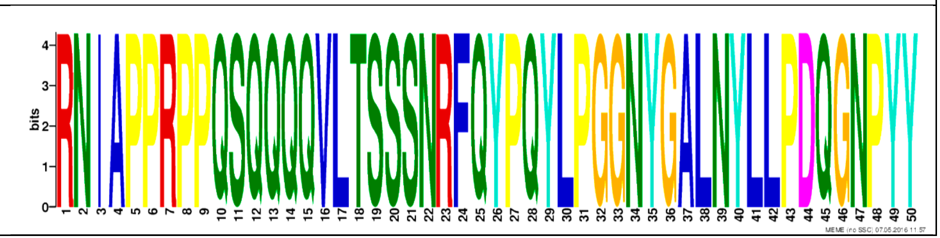

MOTIF-20	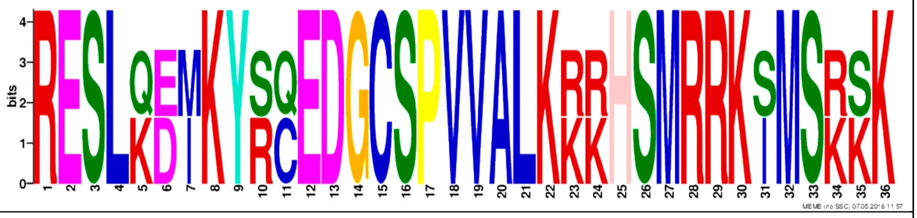
MOTIF-21	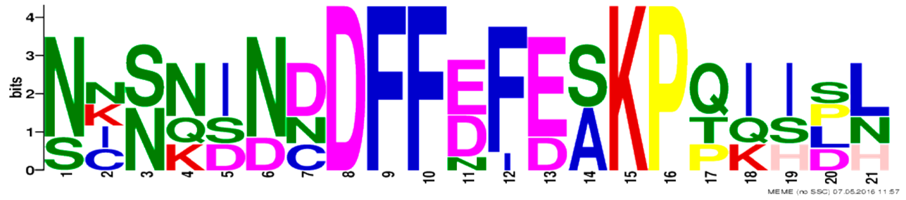
MOTIF-22	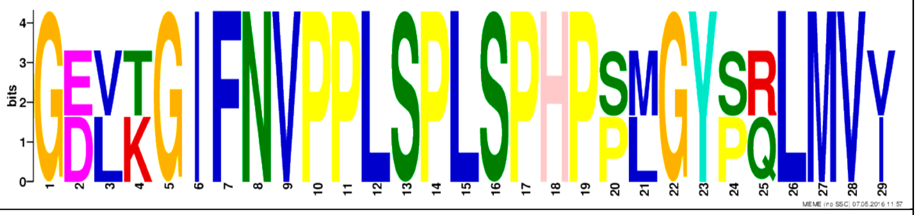
MOTIF-23	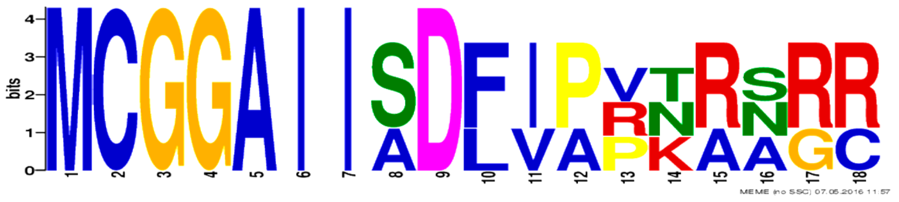
MOTIF-24	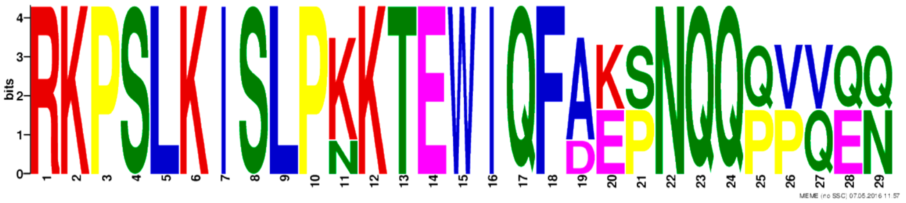
MOTIF-25	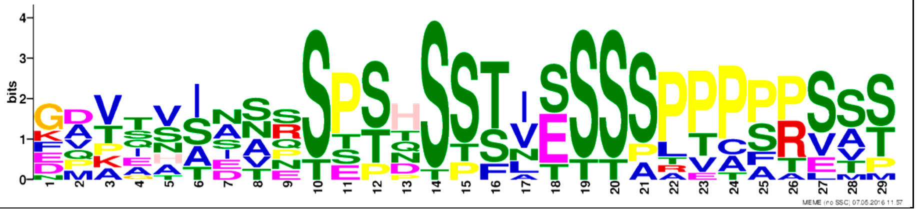

Table S3. The one to one orthologous gene pairs and their corresponding Ka/Ks ratios.

Vf-Transcript-ID	*Vm*-Transcript-ID	Ka	Ks	Ka/Ks	*p*Value
comp181389_c0_seq1	comp129405_c0	0	0.00612149	0	0
comp189934_c0_seq4	comp144341_c0	0	0.0125965	0	0
comp189868_c0_seq1	comp137462_c0	0	0.00433721	0	0
comp187442_c0_seq1	comp162529_c0	0	0.0169105	0	0
comp186047_c0_seq1	comp149797_c0	0	0.00631718	0	0
comp189189_c0_seq2	comp159597_c0	0	0.00514015	0	0
comp192732_c0_seq6	comp166849_c0	0	0.00535543	0	0
comp191512_c0_seq2	comp166307_c0	0	0.0192735	0	0
comp179411_c0_seq1	comp149426_c0	0.00169139	0.101434	0.0166748	5.50004×10^{-19}
comp191827_c0_seq1	comp165608_c0	0.0017379	0.0323087	0.0537903	4.51682×10^{-6}
comp184446_c0_seq1	comp104878_c0	0.00208492	0.0364062	0.0572683	0.00264178
comp174782_c0_seq3	comp144276_c0	0.00201057	0.0215903	0.0931237	0.0330677

comp192264_c0_seq1	comp162707_c0	0.0059197	0.0300008	0.197318	0.00640571
comp194219_c2_seq5	comp162260_c0	0.0142319	0.0661226	0.215235	0.0504604
comp176401_c0_seq4	comp116109_c0	0.00276959	0.00946959	0.292472	0.351771
comp191237_c1_seq13	comp167675_c0	0.00186162	0.00634927	0.293202	0.35172
comp178204_c0_seq1	comp149261_c0	0.0046603	0.0158211	0.294562325	0.0888517
comp180742_c0_seq2	comp151256_c0	0.00668351	0.0188792	0.354015	0.116641
comp195632_c1_seq20	comp165181_c0	0.00339996	0.0087504	0.388549	0.195599
comp184881_c0_seq5	comp159240_c0	0.006005	0.013469	0.445838592	0
comp169343_c0_seq2	comp128319_c0	0.00601339	0.0129221	0.465355	0.340267
comp175038_c0_seq1	comp163543_c0	0.0251589	0.0467847	0.53776	0.30829
comp179927_c0_seq1	comp146876_c0	1.66988	2.90621	0.574591	0.127146
comp184116_c0_seq1	comp165500_c1	0.00186881	0.00315658	0.592036	0.529337
comp173281_c0_seq1	comp143204_c0	0.0490596	0.0818801	0.599164	0.292438
comp181356_c0_seq5	comp128686_c0	0.00421772	0.0059239	0.711983	0.582043
comp181196_c0_seq1	comp150298_c0	0.0176049	0.0244858	0.718984064	0.0337182
comp171489_c1_seq1	comp397454_c0	0.0111181	0.0132456	0.839379	0.391042
comp186790_c1_seq1	comp137242_c0	0.116244	0.121525	0.956542	0.818689
comp171620_c0_seq1	comp129078_c0	0.0103302	0.0103997	0.993317115	0.564387
comp181674_c0_seq1	comp138574_c0	2.21627	2.20093	1.00697	1
comp181660_c0_seq2	comp154195_c0	1.34183	1.2143	1.10502	0.536572
comp184569_c0_seq5	comp154312_c0	0.746161	0.624663	1.1945	0.12609
comp160673_c0_seq2	comp64502_c0	2.02707	1.67002	1.2138	0.485206
comp183962_c0_seq1	comp138429_c0	1.27227	0.949497	1.33994	0.29544
comp181105_c0_seq2	comp137888_c0	0.0880494	0.0573395	1.53558	0.32824
comp178925_c0_seq2	comp139704_c0	2.52379	1.60025	1.57713	0.10624
comp184966_c0_seq2	comp157844_c0	0	0	NA	0
comp180080_c0_seq3	comp124868_c0	0	0	NA	0
comp183897_c0_seq3	comp127938_c0	0	0	NA	0
comp176265_c0_seq1	comp145334_c1	0	0	NA	0
comp158438_c0_seq3	comp110005_c0	0	0	NA	0
comp188688_c0_seq1	comp157176_c0	0	0	NA	0
comp183367_c0_seq5	comp160313_c0	0	0	NA	0
comp160872_c0_seq1	comp173049_c0	0	0	NA	0
comp172871_c0_seq1	comp139267_c0	0	0	NA	0
comp189715_c1_seq1	comp141156_c0	0	0	NA	0
comp192585_c1_seq1	comp155356_c1	0	0	NA	0
comp179276_c0_seq1	comp145295_c0	0	0	NA	0
comp129744_c0_seq1	comp79448_c0	0	0	NA	0
comp174721_c0_seq1	comp122372_c0	0	0	NA	0
comp186054_c0_seq1	comp156517_c1	0	0	NA	0

Table S4. List of specific primer sequences used for real-time PCR analysis.

GeneticName	Primer (5′-3′)	Length
VfAP2/ERF012-F1	ACCGTGTCCTTCAGTAGTTTCG	22
VfAP2/ERF012-R1	GGTCCTGTTAATGGTGCAAAAA	22
VfAP2/ERF022-F1	GTTTCAACTCCTCTTCATCTC	21
VfAP2/ERF022-R1	GATGTTTTTTCTAATGGGTCT	21
VfAP2/ERF043-F1	CTGTTTGTTTCTTTCATGGCGT	22
VfAP2/ERF043-R1	AGCTTCTTCTGGTGTGTCGAATG	23
VfAP2/ERF054-F1	TTCACCAGTAACTGGTTGCCAA	22
VfAP2/ERF054-R1	CCGATGATTATGTGCCGATTAG	22
VfAP2/ERF031-F1	TGATACCGATGCTATTTGG	19
VfAP2/ERF031-R1	CTGCTGCTGTTGTACGTTT	19
VfAP2/ERF027-F1	GTCTAATAATGAACCAGCGGCA	22
VfAP2/ERF027-R1	CAACTCTACTACTGAGCACGGAT	23
VfAP2/ERF047-F1	GGACCTTCGACACAGCTATCGA	22

VfAP2/ERF047-R1	GGTGCGTGTATTCAACTTCCCA	22
VfAP2/ERF058-F1	CTCAGCCCTAGCTCAAGTCATT	22
VfAP2/ERF058-R1	GTGCGTTTGATTGTGGTGGTAT	22
VfAP2/ERF044-F1	CTGCTGCTTTTTTTCTTGCTC	21
VfAP2/ERF044-R1	TTCCGGTGGAAATTGTTAG	19
VfAP2/ERF036-F1	TCTCTCTTGCAAACATGTGTGGT	23
VfAP2/ERF036-R1	GGTAGGGATTGATCTTTATTGCC	23
VfAP2/ERF040-F1	GCCGCCGAAATAAGAGATCC	20
VfAP2/ERF040-R1	GCTTTAGCGCCACGAAAGTC	20
VfAP2/ERF045-F1	GTCAAGAAGATGGTTGCTCAC	21
VfAP2/ERF045-R1	GTTGTTCCAAATAATCAGGG	20
VfAP2/ERF046-F1	AGGTAGTGAAATCTTCAAGGATGG	24
VfAP2/ERF046-R1	TGAAGCTAAACCTCCAAAATCACTC	25
VfAP2/ERF049-F1	TGGAGGCTGGACTGGCTGAT	20
VfAP2/ERF049-R1	AGGGTTTCCTGATAACGGTG	20
VfAP2/ERF051-F1	TTCTTCTTCATCTTTGTCGG	20
VfAP2/ERF051-R1	CATTTCGCATTTCTAACCC	19
VfAP2/ERF052-F1	CATTTTCCTCTCACTTTCCTTC	22
VfAP2/ERF052-R1	CCATGTCTTGTGAGTCATTCTCAT	24
VfAP2/ERF053-F1	ATGATCGTGTACGGTGCTCTTC	22
VfAP2/ERF053-R1	GCGTTACTCTCACTTGCTGCTG	22
VfAP2/ERF055-F1	AACGGTATCTTCTTGAAGACG	21
VfAP2/ERF055-R1	TCAAACTGAACTAACGGAGTC	21
VmAP2/ERF007-F1	ACCCGATATTAACATTTTCGG	21
VmAP2/ERF007-R1	AGTCTTGATTTTGGGGAGGA	20
VmAP2/ERF019-F1	ACCATCTTTGCCTACAACCGCC	22
VmAP2/ERF019-R1	CATTTGCCCCAACTTCTCATCC	22
VmAP2/ERF044-F1	CTGTTTGTTTCTTTCATGGCGT	22
VmAP2/ERF044-R1	AGCTTCTTCTGGTGTGTCGAATG	23
VmAP2/ERF056-F1	TTCACCAGTAACTGGTTGCCAA	22
VmAP2/ERF056-R1	CCGATGATTATGTGCCGATTAG	22
VmAP2/ERF032-F1	CAATACCAGAGGCAAAACCACT	22
VmAP2/ERF032-R1	GATGACCTGCTTCTCCAAATAG	22
VmAP2/ERF027-F1	GACTTCCTTCTTCCTCGCAAACC	23
VmAP2/ERF027-R1	AAACAACCACACCCTTGACGCT	22
VmAP2/ERF050-F1	GATTGTAGAGGAGCAAGCGAA	21
VmAP2/ERF050-R1	TGGATGAGGAGATAACGGAGAC	22
VmAP2/ERF062-F1	CACACAAAGACAGATCGACGTAA	23
VmAP2/ERF062-R1	GGCTGAAAAAAGAGGGGAGAAT	22
VmAP2/ERF046-F1	CTGCTGCTTTTTTTCTTGCTC	21
VmAP2/ERF046-R1	TTCCGGTGGAAATTGTTAG	19
VmAP2/ERF036-F1	GGGAAATGGGCTGCTGAGAT	20
VmAP2/ERF036-R1	ACTGCTGGTGGTTGTTGTGA	20
VmAP2/ERF039-F1	AAACCATCACCACACAACCACC	22
VmAP2/ERF039-R1	CTTTCGGAGCCATTTCGCA	19
VmAP2/ERF047-F1	CAACAAGGCATGGAATTAGA	20
VmAP2/ERF047-R1	TTAGAGCAACAACAGGTGAGC	21
VmAP2/ERF049-F1	CTTCCTTGGCTCTTCCTATTTC	22
VmAP2/ERF049-R1	GATGATGAACAAGCTACAGGTGA	23
VmAP2/ERF052-F1	TGGAGGCTGGACTGGCTGAT	20
VmAP2/ERF052-R1	AGGGTTTCCTGATAACGGTG	20
VmAP2/ERF059-F1	CCCGAAGTACAGCTGCCAGAGA	22

VmAP2/ERF059-R1	CTAACCAAACCCGAGCCCCAT	21
VmAP2/ERF054-F1	GCAACATCAACAACTCATCCTC	22
VmAP2/ERF054-R1	CTTCCGTTATCGTGCTACTCTC	22
VmAP2/ERF055-F1	TAGAGCAGCCTAATAAAAAGA	21
VmAP2/ERF055-R1	CCATTATCCAAAGTGAATCAAACA	24
VmAP2/ERF057-F1	TCAAACTGAACTAACGGAGTCG	22
VmAP2/ERF057-R1	GCGGTATCTTCTCGAAGACGA	21

Table S5. Pearson correlation (*r*) between transcriptome data and qRT-PCR result of selected orthologous genes in *V. fordii* and *V. montana.*

#	GeneticName	*r*
1	*VfAP2/ERF012*	0.875
2	*VfAP2/ERF022*	0.574
3	*VfAP2/ERF043*	0.997
4	*VfAP2/ERF054*	0.996
5	*VfAP2/ERF031*	0.986
6	*VfAP2/ERF027*	0.997
7	*VfAP2/ERF047*	0.718
8	*VfAP2/ERF058*	0.685
9	*VfAP2/ERF044*	0.825
10	*VfAP2/ERF045*	0.048
11	*VfAP2/ERF053*	0.732
12	*VmAP2/ERF007*	0.999
13	*VmAP2/ERF019*	0.978
14	*VmAP2/ERF044*	0.994
15	*VmAP2/ERF056*	0.993
16	*VmAP2/ERF032*	0.96
17	*VmAP2/ERF027*	0.361
18	*VmAP2/ERF050*	0.994
19	*VmAP2/ERF062*	0.947
20	*VmAP2/ERF046*	0.337
21	*VmAP2/ERF047*	0.978
22	*VmAP2/ERF055*	0.968
